# Genome mining for ribosomally synthesized and post-translationally modified peptides (RiPPs) in anaerobic bacteria

**DOI:** 10.1186/1471-2164-15-983

**Published:** 2014-11-18

**Authors:** Anne-Catrin Letzel, Sacha J Pidot, Christian Hertweck

**Affiliations:** Leibniz Institute for Natural Product Research and Infection Biology HKI, Beutenbergstr. 11a, Jena, 07745 Germany; Doherty Institute, The University of Melbourne, Parkville, Melbourne, Victoria 3010 Australia; Chair of Natural Product Chemistry, Friedrich Schiller University, Jena, 07743 Germany

**Keywords:** Genome mining, RiPP, Anaerobic bacteria, Clostridia, Genomics, Natural product biosynthesis

## Abstract

**Background:**

Ribosomally synthesized and post-translationally modified peptides (RiPPs) are a diverse group of biologically active bacterial molecules. Due to the conserved genomic arrangement of many of the genes involved in their synthesis, these secondary metabolite biosynthetic pathways can be predicted from genome sequence data. To date, however, despite the myriad of sequenced genomes covering many branches of the bacterial phylogenetic tree, such an analysis for a broader group of bacteria like anaerobes has not been attempted.

**Results:**

We investigated a collection of 211 complete and published genomes, focusing on anaerobic bacteria, whose potential to encode RiPPs is relatively unknown. We showed that the presence of RiPP-genes is widespread among anaerobic representatives of the phyla Actinobacteria, Proteobacteria and Firmicutes and that, collectively, anaerobes possess the ability to synthesize a broad variety of different RiPP classes. More than 25% of anaerobes are capable of producing RiPPs either alone or in conjunction with other secondary metabolites, such as polyketides or non-ribosomal peptides.

**Conclusion:**

Amongst the analyzed genomes, several gene clusters encode uncharacterized RiPPs, whilst others show similarity with known RiPPs. These include a number of potential class II lanthipeptides; head-to-tail cyclized peptides and lactococcin 972-like RiPP. This study presents further evidence in support of anaerobic bacteria as an untapped natural products reservoir.

**Electronic supplementary material:**

The online version of this article (doi:10.1186/1471-2164-15-983) contains supplementary material, which is available to authorized users.

## Background

The increasing number of multiresistant bacteria pose a constant challenge for medicine and dictate the necessity of developing new antimicrobial compounds to treat life-threatening infections. Ribosomally synthesized and post-translationally modified peptides (RiPPs) are a promising addition to antibiotics biosynthesized via polyketide or non-ribosomal pathways. As antimicrobial agents this group of compounds often possess a narrow activity spectrum, most often targeting near relatives of the producing organism, although some broader spectrum RiPPs have been identified [1–3]. Their limited range of activity makes RiPPs potential targets for clinical applications as they can avoid the off-target effects seen with broad spectrum antibiotic agents, which can disturb the normal flora and open the door to undesired secondary infections by resistant organisms [3]. Although their target organisms may be highly specific, RiPPs have been shown to interrupt a variety of cellular processes, including the disruption of DNA, RNA or protein biosynthesis, although they commonly form pores in cell membranes by either targeting lipid II, a cell wall building block, or by direct pore formation via insertion into the cell wall [3]. As the targets of these compounds are conserved amongst many bacteria and are not subject to heavy modification, the potential for the development of resistance against RiPPs is significantly diminished [3, 4].

Despite the fact that RiPPs cover a diverse range of structural classes, they all follow a simple biosynthetic logic: a precursor peptide consisting of an *N*-terminal leader sequence and a *C*-terminal core sequence, encoded by a single gene is translated, the leader sequence is removed by a series of transporters, peptidases or a combination of both, and the remaining active peptide moiety is further processed by other enzymes, often encoded by genes within close proximity to the precursor gene [1, 2, 5].

The genetic basis for the production of many RiPP classes is well understood, and in most cases, gene content and structure is conserved amongst different arms of the bacterial phylogenetic tree. As such, comparison of well characterized biosynthetic genes or gene clusters against new genome sequences can identify putative RiPPs and in some cases, even the structure of the target metabolite can be predicted [6]. This "genome mining" approach allows for the discovery of potentially novel natural products in a completely culture independent fashion, with the potential to reduce the rediscovery rate of known molecules. Furthermore, genome mining has expanded the definition of exactly what constitutes a secondary metabolite producer and has revealed that the biosynthetic potential of many microorganisms has been widely underestimated [7–9]. Amongst these newly identified producers lie the anaerobic bacteria, a group that were believed to be incapable of producing secondary metabolites, as life without oxygen was presumed to not provide the required energy for the complex biosynthesis of antibiotics [10]. These "neglected" bacteria include those that are known to produce highly toxic peptides (botulinum toxin, tetanus toxin), and more recently several species have been identified as the source of novel natural products [8, 11–13]. An extensive investigation of 211 genomes of anaerobic bacteria for the presence of polyketide synthase (PKS) or non-ribosomal peptide synthetase (NRPS) encoding genes revealed a much larger potential than previously suspected and many of these PKS or NRPS loci appeared to be novel, with limited homology to previously characterized gene clusters [8]. Furthermore, it showed that certain genera have a predisposition towards increased secondary metabolite potential (such as members of the phyla Proteobacteria and Firmicutes) and that the natural habitat of the organisms seems to play an important role – isolates from environmental strains (soil, mud) often contain up to three times more genes for secondary metabolite biosynthesis than all other habitats combined. In particular, the clostridia were shown to be a potential treasure trove of novel secondary metabolites, which the isolation of the novel antibiotics closthioamide and clostrubin have recently confirmed [13, 14].

Despite the recent investigation of anaerobes for their potential to produce polyketide or non-ribosomal peptide metabolites [8], little is known about their ability to produce RiPPs. As anaerobes have been shown to possess a wealth of novel biosynthetic gene clusters, this suggests that there is also the potential to identify novel RiPP genetic loci amongst these organisms. This may, in turn, lead to the discovery of novel antimicrobial compounds to treat multi-drug resistant infections. Here we present an in-depth investigation of RiPP-encoding genes within the genomes of 211 anaerobic bacteria. As the nomenclature for RiPPs was redefined in 2013, with the support of many in the natural products community, we have chosen to follow these recommendations here, and have placed a size limit of 10 kDa for inclusion as a potential RiPP [1]. We have used a variety of bioinformatic tools in our analysis, including antiSMASH [15, 16], Bagel and bactibase database screening [17, 18], and BLAST searches to identify and predict the presence of RiPP gene clusters. Here we have shown that anaerobes have the potential to produce a variety of different RiPPs and that there is tendency towards the presence of RiPP biosynthetic gene clusters within those that already possess genetic loci for other secondary metabolites.

## Results and discussion

### General features of anaerobe genomes with respect to RiPPs

**Figure 1:**
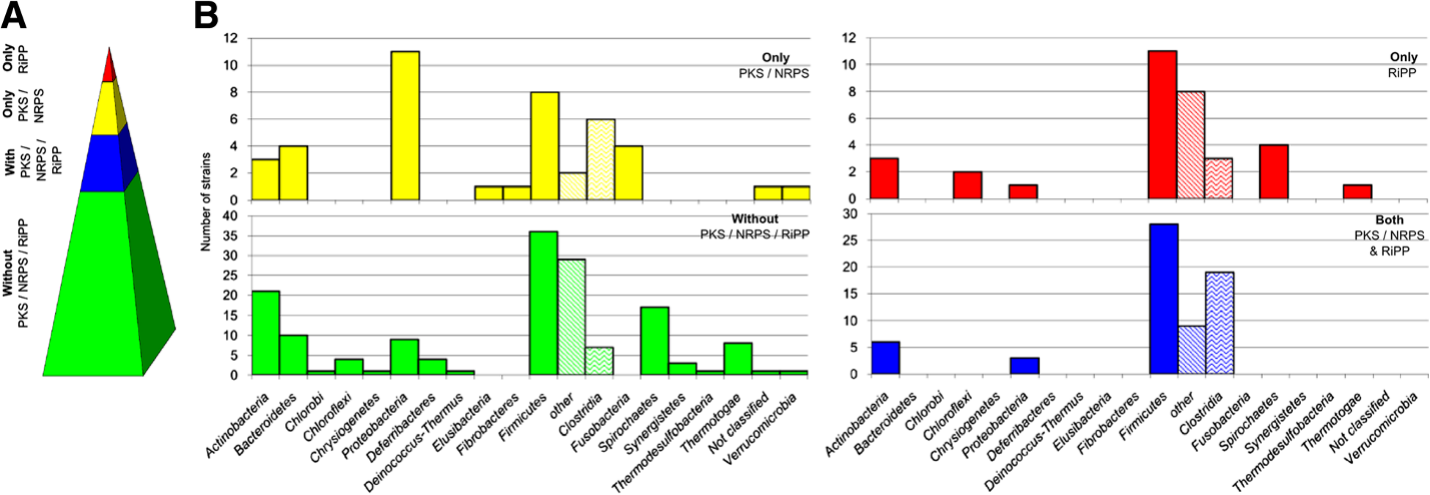
**Potential of anaerobic bacteria for PKS/NRPS**/**RiPP production and distribution among different phyla. A** Distribution of genes for secondary metabolite production; percentage of strains containing: no PKS/NRPS/RiPP genes (green); both PKS/NRPS and RiPP (blue); only PKS/NRPS (yellow); only RiPP (red) **B** Distribution of secondary metabolite containing strains according to phyla and ability for secondary metabolite production (no PKS/NRPS/RiPP genes (green); both PKS/NRPS and RiPP genes (blue); only PKS/NRPS genes (yellow); only RiPP genes (red)). *Firmicutes* are additionally divided into *Clostridia* and others.

To survey the diversity of RiPPs we have undertaken a bioinformatic investigation of 211 complete and published anaerobe genomes for the presence of RiPP genes and gene clusters. Of note is the fact that anaerobes are a potential source of RiPPs, with >25% of currently sequenced anaerobe genomes encoding at least one or more RiPP classes (Table 1). It appears as though the RiPP biosynthetic gene clusters are more likely to be found in strains that possess other secondary metabolite biosynthetic gene loci, with only 10.4% of analyzed genomes containing only RiPP-encoding genes. However, these trends may only be predictable for the phyla Firmicutes, Actinobacteria, Bacteriodetes, Proteobacteria and Spirochaetes*,* which comprise a sufficient number of genomes for a representative analysis (Table 1, Figure 1). To what extent the present results also represent a general trend for the other phyla is difficult to estimate and more genomes of these phyla are required. The combination of PKS/NRPS and RiPPs appears to be limited to the phyla Actinobacteria, Proteobacteria and Firmicutes, confirming previous reports in aerobic organisms [1]. Notably, RiPP biosynthetic gene clusters were not identified in any anaerobes from the phylum Bacteriodetes, although aerobes from this phylum have been shown to possess lanthipeptide gene clusters [1]. In contrast to the situation with PKS/NRPS gene clusters, which are absent in Spirochaetes genomes, a small number of these organisms appear capable of producing RiPPs (Table 1, Figure 1). As is the case with PKS/NRPS biosynthetic gene clusters, of the sequenced genomes in our analysis, the Firmicutes appear to contain the highest percentage of RiPP producers, with approximately 75% of the *Clostridium* species analyzed being capable of producing PKS/NRPS or RiPPs.

**Table 1:** **Distribution of the presence of PKS/NRPS/RiPPs according to phyla**

Phylum	Number of strains	Number of strains only RiPP	Number of strains only PKS/ NRPS	Number of strains without PKS/ NRPS/ RiPP	Number of strains both PKS/ NRPS and RiPP
Actinobacteria	33	3(9.1%)	3(9.1%)	21(63.6%)	6(18.2%)
Bacteroidetes	14	0	4(28.6%)	10(71.4%)	0
Chlorobi	1	0	0	1(100%)	0
Chloroflexi	6	2(33.3%)	0	4(66.7%)	0
Chrysiogenetes	1	0	0	1(100%)	0
Proteobacteria	24	1(4.2%)	11(45.8%)	9(37.5%)	3(12.5%)
Deferribacteres	4	0	0	4(100%)	0
Deinococcus-Thermus	1	0	0	1(100%)	0
Elusibacteria	1	0	1(100%)	0	0
Fibrobacteres	1	0	1(100%)	0	0
Firmicutes	83	11(13.3%)	8(9.6%)	36(43.4%)	28(33.7%)
Other	48	8(16.7%)	2(4.2%)	29 (60.4%)	9(18.7%)
*Clostridium*	35	3 (8.6%)	6(17.2%)	7(20.0%)	19(54.2%)
Fusobacteria	4	0	4(100%)	0	0
Spirochaetes	21	4(19.0%)	0	17(81.0%)	0
Synergistetes	3	0	0	3(100%)	0
Thermodesulfobacteria	1	0	0	1(100%)	0
Thermotogae	9	1(11.1%)	0	8(88.9%)	0
Verrucomicrobia	2	0	1(50%)	1(50%)	0
Not classified	2	0	1(50%)	1(50%)	0
**Total**	**211**	**22**(10.4%)	**34**(16.1%)	**118**(55.9%)	**37**(17.6%)

**Figure 2:**
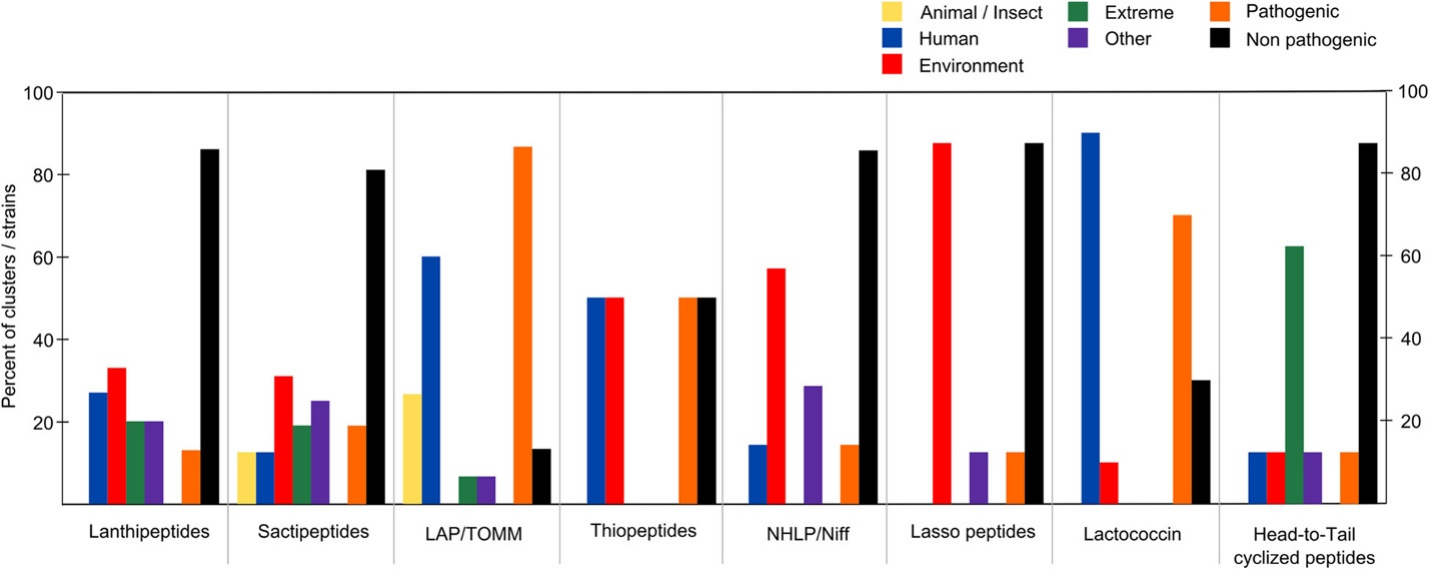
**Distribution of different RiPP biosynthetic gene clusters by habitat and pathogenicity.** Animal (yellow); human (blue), environment (soil/mud) (red); extreme (green); other (purple); pathogenic (orange); non- pathogenic (black).

When it comes to the kind of RiPPs which are produced by the respective strains lanthipeptides, sactipeptides and linear azol(in)e-containing peptides (LAP) are the most common types (each group of RiPPs is explained in further detail below). LAP- and lactococcin-like genes are present predominantly in human pathogenic strains, whilst strains from extreme environments tend to contain DNA encoding head-to-tail cyclized peptides, as well as lanthipeptides and sactipeptides (Table 2, Figure 2). Lasso peptide biosynthetic loci appear to be mainly contained within non-pathogen genomes, and the lanthipeptides also appear to follow a similar distribution. Proteobacteria predominantly contain lasso peptide gene clusters and these are also more common in non-clostridia Firmicutes as well as head-to-tail cyclized peptides (Table 2, Figure 2).

**Table 2:** **Distribution of different RiPPs according to phylum**

Phylum	Lanthi- peptides	Sacti- peptides	LAP/ TOMM	Thio- peptides	NHLP/ Niff	Lasso peptides	Lacto- coccins	Head- to- Tail cyclized peptides	Total
Actinobacteria	3			1	1		8		**13**
Bacteroidetes									
Chlorobi									
Chloroflexi								2	**2**
Chrysiogenetes									
Proteobacteria		1			1	3			**5**
Deferribacteres									
Deinococcus-Thermus									
Elusibacteria									
Fibrobacteres									
Firmicutes	12	14	11	1	5	5	2	6	**56**
Other	3	6	1		5	4	1	5	**25**
*Clostridium*	9	8	10	1		1	1	1	**31**
Fusobacteria									
Spirochaetes			4						**4**
Synergistetes									
Thermodesulfobacteria									
Thermotogae		1							**1**
Verrucomicrobia									
Not classified									
**Total**	**15**	**16**	**15**	**2**	**7**	**8**	**10**	**8**	**81**

Although the focus here is on RiPP classes, several other peptides with potential antimicrobial activity, such as holins, linocins or peptidases were also identified. However, as their predicted size is much bigger than for the RiPPs, they were excluded from the following analyses.

### Lanthipeptides

**Figure 3:**
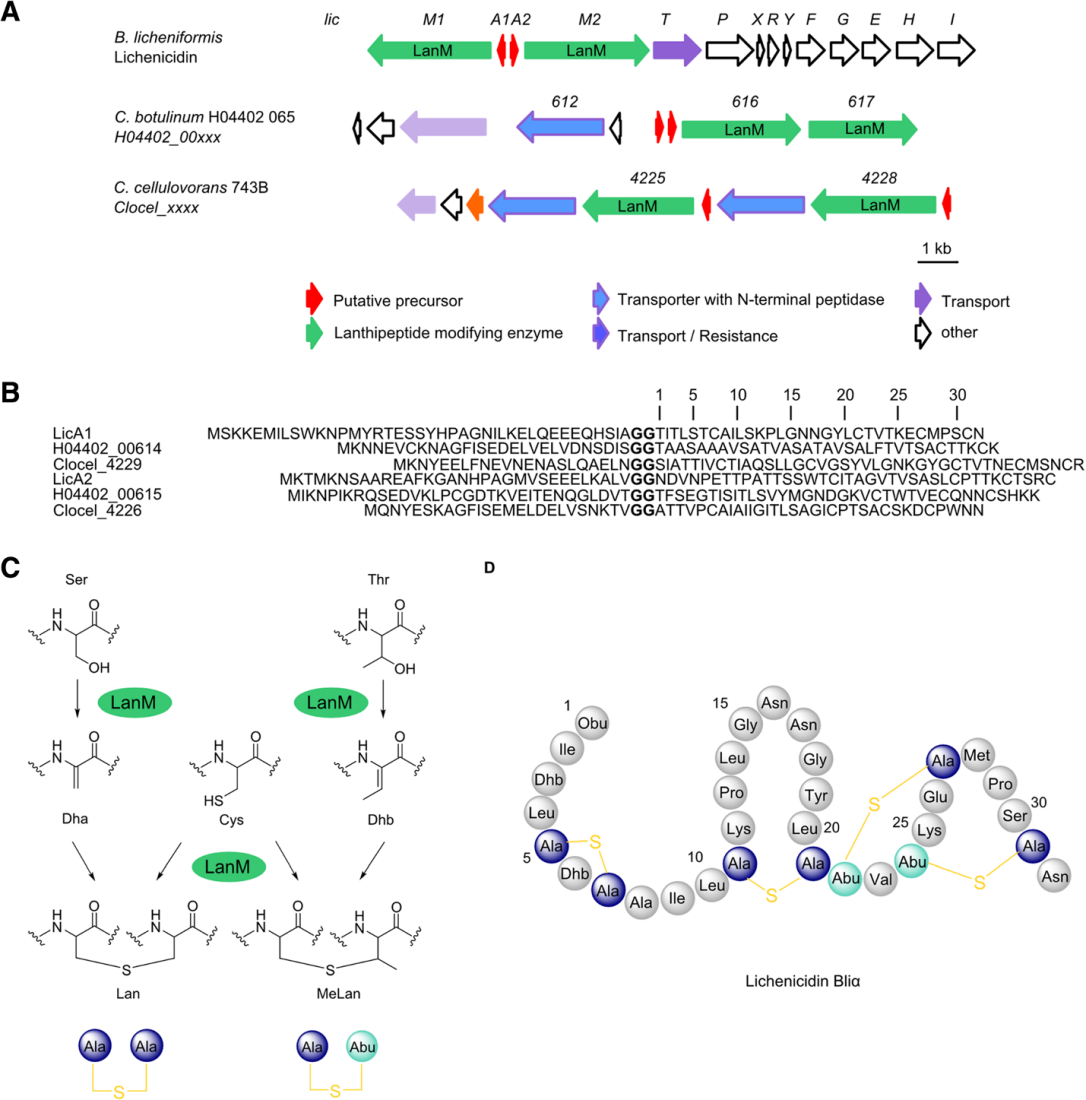
**Lichenicidin**-**like lanthipeptides. A** Lichenicidin biosynthetic gene cluster (*lic*) of *B. licheniformis* in comparison to putative lichenicidin gene clusters of *C. botulinum* H04402 065 and *C. cellulovorans* 743B; Numbers represent the locus tag for each gene within the genome sequence of each organism. **B** Comparison of lichenicidin peptide precursors (LicA1 and LicA2) and the putative precursor peptides of *C. botulinum* H04402 065 (H04402_00614 and H04402_00615) and *C. cellulovorans* 743B (Clocel_4229 and Clocel_4226); Glycine-Glycine motif indicates the cleavage site of leader sequence and core peptide (bold) **C** Formation of lanthionine (lan) and methyl-lanthionine (me-lan) moieties by dehydration of serine/threonine residues to dehydroalanine (dha) / dehydrobutyrine (dhb) and subsequent cyclization with a cysteine residue catalyzed by LanM **D** Amino acid structure of lichenicidin α-subunit (Bliα).

The lanthipeptides are defined by the presence of the non-proteinogenic amino acids lanthionine and 3-methyllanthionine, which are crosslinked via a thioether linkage at their β-carbon atoms [1, 2, 5, 19, 20]. The best-known and characterized lanthipeptide is nisin, which was first reported in 1928, although its structure was only finally elucidated in the 1970s [9, 10]. The biosynthetic genes for nisin had to wait until the late 1980s to be uncovered, and since this time many *lan* biosynthetic loci have been identified. The synthesis of the unusual lanthionine and 3-methyllanthionine residues occurs by dehydration of serine and threonine to dehydroalanine (dha) and dehydrobutyrine (dhb), respectively, via phosphorylated intermediates, which subsequently undergo a Michael-type addition (cyclization) with a cysteine residue [1, 5, 19] (Figure 3).

Based on the enzymes that are responsible for the post-translational modifications of the precursor peptides, lanthipetides are divided into four different groups. Class I lanthipeptides possess two distinct enzymes that carry out the dehydration (LanB) and cyclization (LanC). Class II-IV lanthipeptide are modified by multifunctional enzymes. Class II lanthipeptides possess a bifunctional enzyme with a *N*-terminal dehydratation (LanM- similar to LanB), and a *C*-terminal cyclisation domain (similar LanC). In the case of class III and IV lanthipeptides the modifications are carried out by a specific tri functional enzymes LanKC and LanL, respectively, which consist of an *N*-terminal phosphoserine- or phosphothreoninelyase, a central kinase and a *C*-terminal cyclisation domain [1, 2, 5, 19]. Class III lanthipeptides may also contain another non-proteinogenic amino acid – labionin [21–23]. Labionin follows a similar biosynthetic route to lanthionine, with cyclodehydration of a cysteine and two serine residues, which then react in a second Michael addition with another dehydroalanine [21–23]. Due to the strong conservation of these modifying enzymes they can be used for genome mining approaches [24, 25]. To date, characterized labionin-containing lanthipeptides are the labyrinthopeptide [21], erythreapeptin [26], avermipeptin [26], griseopeptin [26], catenulipeptin [27] and NAI112 [23]. The activity spectrum of lanthipeptides are mostly limited to Gram-positive bacteria and the mode of action is often associated with the disturbance of cell wall biosynthesis and pore formation [19], however, biosurfactant lanthipeptides have also been identified [28, 29].

**Figure 4:**
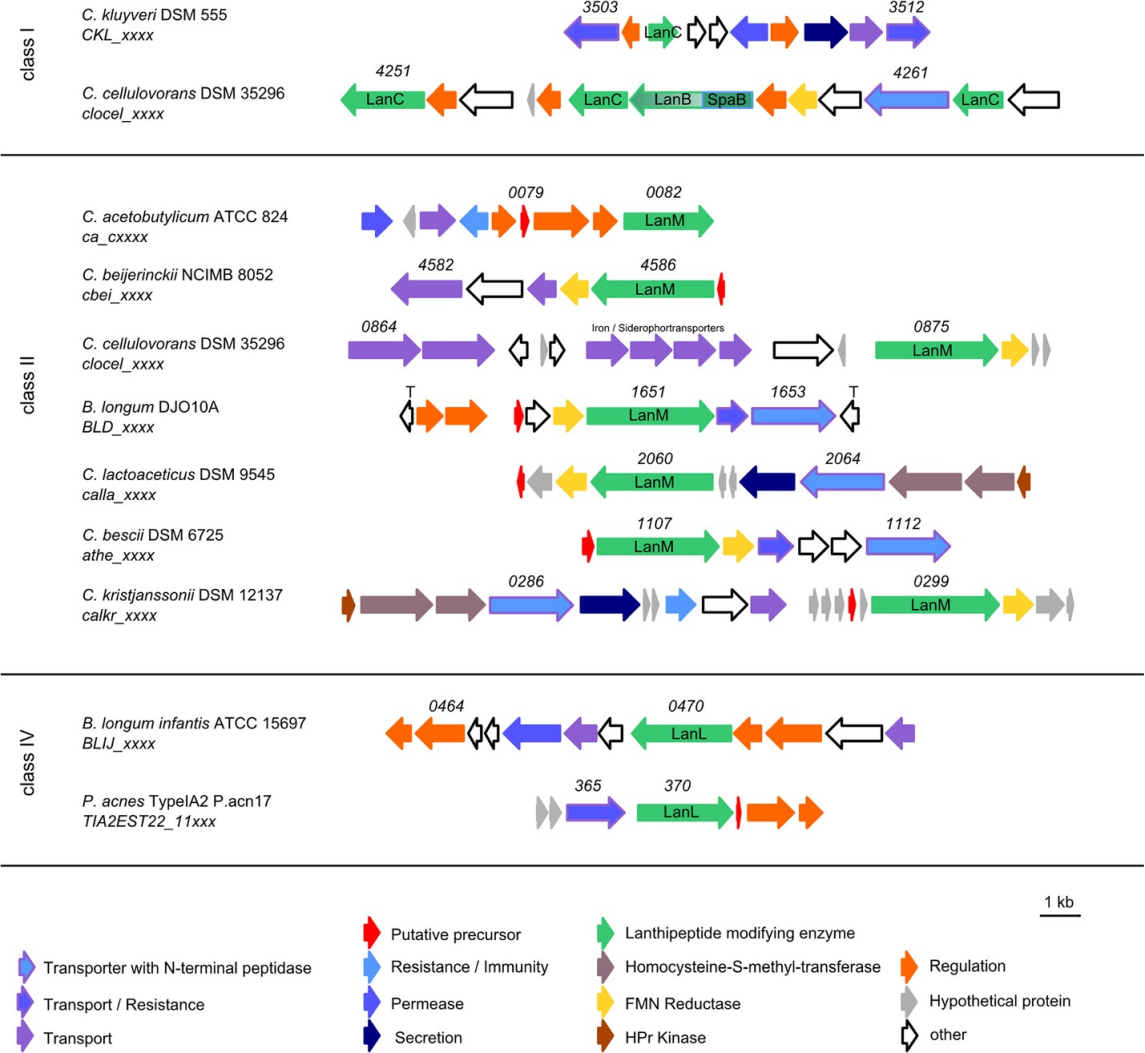
**Detected putative lanthipeptide gene clusters sorted by similar biosynthetic origin.** Numbers represent the locus tag for each gene within the genome sequence of each organism.

In total 15 putative lanthipeptide biosynthetic gene clusters were detected in the analysed bacterial genomes, with class II lanthipeptides being the most common (Table 3, Figures 3 and 4). The lanthipeptides were once thought to be restricted to the Actinobacteria and Firmicutes phyla and in terms of anaerobes, this also appears to be the case [1]. Whilst some predicted lanthipeptide biosynthetic gene clusters exhibit similarity to previously characterized 2-component lanthipeptides, such as those for lichenicidin VK21 (*Bacillus licheniformis* VK21) [30] or lichenicidin (*Bacillus licheniformis* DSM 13 (ATCC 14580)), the remaining lanthipeptide gene clusters appear to be unique. The lichenicidins consist of two single peptides which gain their full activity only in combination [30] and they are encoded by two different precursor peptides as well as modified by two separate LanM enzymes. The leader sequence is removed during the transport out of the cell by the bifunctional enzyme LanT (transporter with *N*-terminal protease) [31].

**Table 3:** **Detected putative lanthipeptide gene clusters**

	Phylum	Lanthipeptide class	Locus tag of the lanthipeptide modifying enzyme Lan	Similar to ^†^	Reference ^#^
*Clostridium cellulovorans* 743B, ATCC 35296	Firmicutes	I	*Clocel_4251*		
			*Clocel_4256*		
			*Clocel_4262*		
*Clostridium kluyveri* DSM 555	Firmicutes	I	*CKL_3505*		
*Bifidobacterium longum* DJO10A	Actino-bacteria	II	*BLD_1651*		[25]
*Clostridium acetobutylicum* ATCC 824	Fimicutes	II	*CA_C0082*		
*Clostridium acetobutylicum* DSM 1731	Fimicutes	II	*SMB_G0083*		
*Clostridium acetobutylicum* EA 2018	Fimicutes	II	*CEA_G0073*		
*Clostridium beijerinckii* NCIMB 8052	Fimicutes	II	*Cbei_4586*		[25]
*Clostridium botulinum* H04402 065	Fimicutes	II	*H04402_00616 H04402_00617*	lichenicidin	
*Clostridium cellulovorans* 743B, ATCC 35296	Fimicutes	II	*Clocel_0875*	lichenicidin	[32]
		II	*Clocel_ 4225*		
			*Clocel_4228*		
*Caldicellulosiruptor lactoaceticus* 6A, DSM 9545	Fimicutes	II	*Calla_2060*		
*Caldicellulosiruptor bescii* Z-1320, DSM 6725	Fimicutes	II	*Athe_1107*	lichenicidin	[32]
*Caldicellulosiruptor kristjanssonii* 177R1B, DSM 12137	Fimicutes	II	*Calkr_0299*		
*Bifidobacterium longum infantis* JCM 1222, ATCC 15697	Actino-bacteria	IV	*BLIJ_0470*		
*Propionibacterium acnes* TypeIA2 P.acn17	Actino-bacteria	IV	*TIA2EST22_11370*		

^†^Cluster shows similarities to characterized RiPP cluster; ^#^Cluster was previously detected by genome mining approaches.

Similar to the lichenicidin gene cluster, two precursor peptide encoding genes (A1, A2) and two LanM (M1, M2) encoding genes were detected in the genomes of *Clostridium botulinum* H04402 065 and *Clostridium cellulovorans* 743B (Figure 3). The arrangement of the genes is different in the respective clusters, but all the necessary core proteins appear to be encoded. The lichenicidin gene cluster, however, possesses a number of genes for immunity, which were not detected in the orthologous gene clusters in the clostridia. However, the heterologous expression of the *B. licheniformis* lichenicidin gene cluster in *E. coli* has shown that the immunity genes are not necessary for production of the lantibiotic, suggesting that the clostridial lichenicidin-like gene clusters may also be capable of producing an active lantibiotic [33]. It is also possible that the immunity gene(s) are located elsewhere in the genome. BLAST analysis of the putative precursor peptides of *Caldicellulosiruptor bescii* Z-1320 also showed similarities to lichenicidin, but only one precursor peptide and one modifying LanM protein are encoded in this cluster (Figure 4).

### Sactipeptides

**Figure 5:**
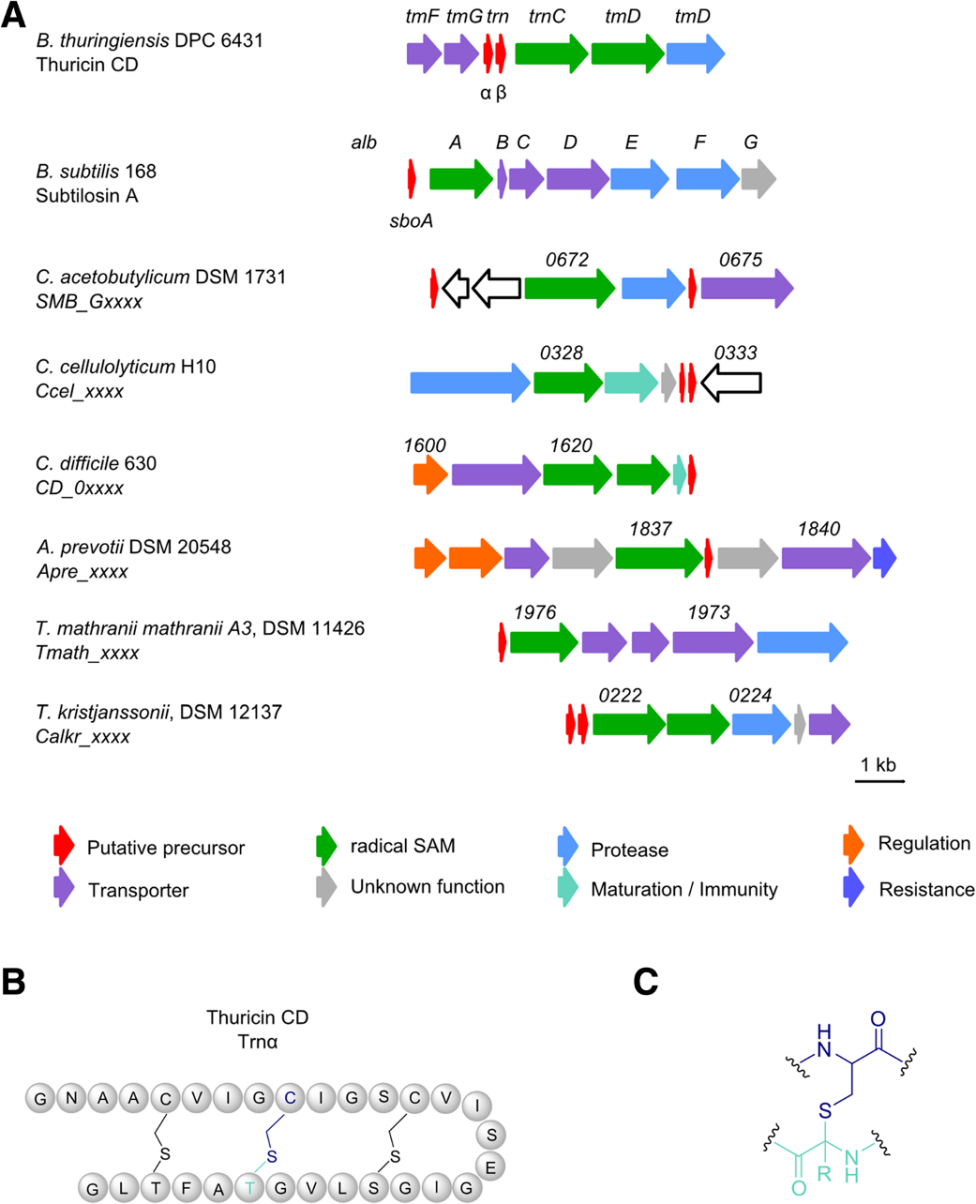
**Detected putative sactipeptides. A** Thuricidin CD gene cluster (*tm*) of *B. thuringiensis* DPC 6431 and subtilosin A gene cluster (*alb*) of *B. subtilis* 168 in comparison to detected putative sactipeptide gene clusters; Numbers represent the locus tag for each gene within the genome sequence of each organism. **B** Amino acid structure of thuricin CD α-subunit (Trnα) **C** Characteristic sulfur bridge between a cysteine residue and the α-carbon of another residue in sactipeptides.

Sactipeptides or sactibiotics (sulphur to alpha-carbon antibiotic) are peptides in which a sulfur bridge is post-translationally formed between a cysteine residue and the α-carbon of another residue (Figure 5B & C), in contrast to lanthipeptides where the sulfur bridge is installed via the β-carbon [1, 34]. The sulfur linkage is introduced via a special radical SAM enzyme whose gene is co-localized in all sactipetide gene clusters and can be used for genome mining approaches [1, 35–37]. Several sactipeptides have so far been elucidated, all from *Bacillus* species, and include subtilosin A (*B. subtilis*, hemolytic) [38, 39], thuricin CD with its components Trn-α and Trn-β (*B. thuringiensis*, anticlostridial) [40], thurincin H (*B. thuringiensis*) [41] and the sporulation killing factor (SKF) (*B. subtilis*) [42]. Approximately 0.5% of the total protein content of anaerobic bacteria is represented by highly diverse radical SAM enzymes [43], and using putative radical SAM enzymes as a means of identifying sactipeptide loci returned a large number of enzymes putatively involved in RiPP formation. A similar approach was previously taken by Murphy *et al.*, using the radical SAM enzyme of the thuricin CD gene cluster as BLAST template, which identified several thuricin CD-like biosynthetic gene clusters, including several in anaerobic bacteria [37].

In this study many putative sactipeptide like gene clusters were obtained by using BAGEL database in a similar fashion to those reported previously [37]. Screening of the genes surrounding the encoded radical SAM proteins for sactipeptide like accessory genes (such as transporters and other proteins related to peptide maturation or secretion) led to the exclusion of many putative gene clusters, with those remaining listed in Table 4. Several of the gene clusters showed similarities to thuricin CD (Figure 5A) as mentioned above, however, the gene organization and number of precursor peptides differ between strains. It appears that the number of radical SAM enzymes encoded within a gene cluster correlates with the number of putative precursor peptides, except in case of *Clostridium cellulolyticum* H10 where only one radical SAM per two precursor peptides and *Clostridium difficile* 630 where two radical SAM enzymes per precursor peptide are encoded (Figure 5A).

**Table 4:** **Detected putative sactipeptide gene cluster**

	Phylum	Locus tag of radical SAM	Similar to ^†^	Reference ^#^
*Clostridium acetobutylicum* DSM 1731	Firmicutes	*SMB_G0673*		
*Clostridium acetobutylicum* ATCC 824	Firmicutes	*CA_C0658*		
*Clostridium acetobutylicum* EA 2018	Firmicutes	*CEA_G0670*		
*Clostridium cellulolyticum* H10	Firmicutes	*Ccel_0328*	thuricin	[37]
*Clostridium difficile* 630	Firmicutes	*CD630_1620*	thuricin	[37]
*Clostridium kluyveri* DSM 555	Firmicutes	*CKL_0375*		
*Clostridium lentocellum* RHM5, DSM 5427	Firmicutes	*Clole_0089*		
*Clostridium thermocellum* ATCC 27405	Firmicutes	*Cthe_1695*		
*Anaerococcus prevotii* PC1, DSM 20548	Firmicutes	*Apre_1837*	thuricin	[37]
*Ruminococcus albus* 7, ATCC 27210	Firmicutes	*Rumal_0164*		
*Syntrophobotulus glycolicus* FIGlyR, DSM 8271	Firmicutes	*Sgly_1407*		
*Thermoanaerobacter mathranii mathranii* A3*,* DSM1142	Firmicutes	*Tmath_1976*	thuricin	[37]
*Caldicellulosiruptor kristjanssonii* 177R1B, DSM 12137	Firmicutes	*Calkr_0222*	thuricin	[37]
*Halothermothrix orenii* H 168	Firmicutes	*Hore_04320*		
*Desulfobacca acetoxidans* ASRB2, DSM 111069	δ- Proteobacteria	*Desac_0107*		
*Thermosipho melanesiensis* BI429	Thermotogae	*Tmel_0409*		

^†^Cluster shows similarities to characterized RiPP cluster; ^#^Cluster was previously detected by genome mining approaches.

### Linear azol(in)e- containing peptides (LAP)

**Figure 6:**
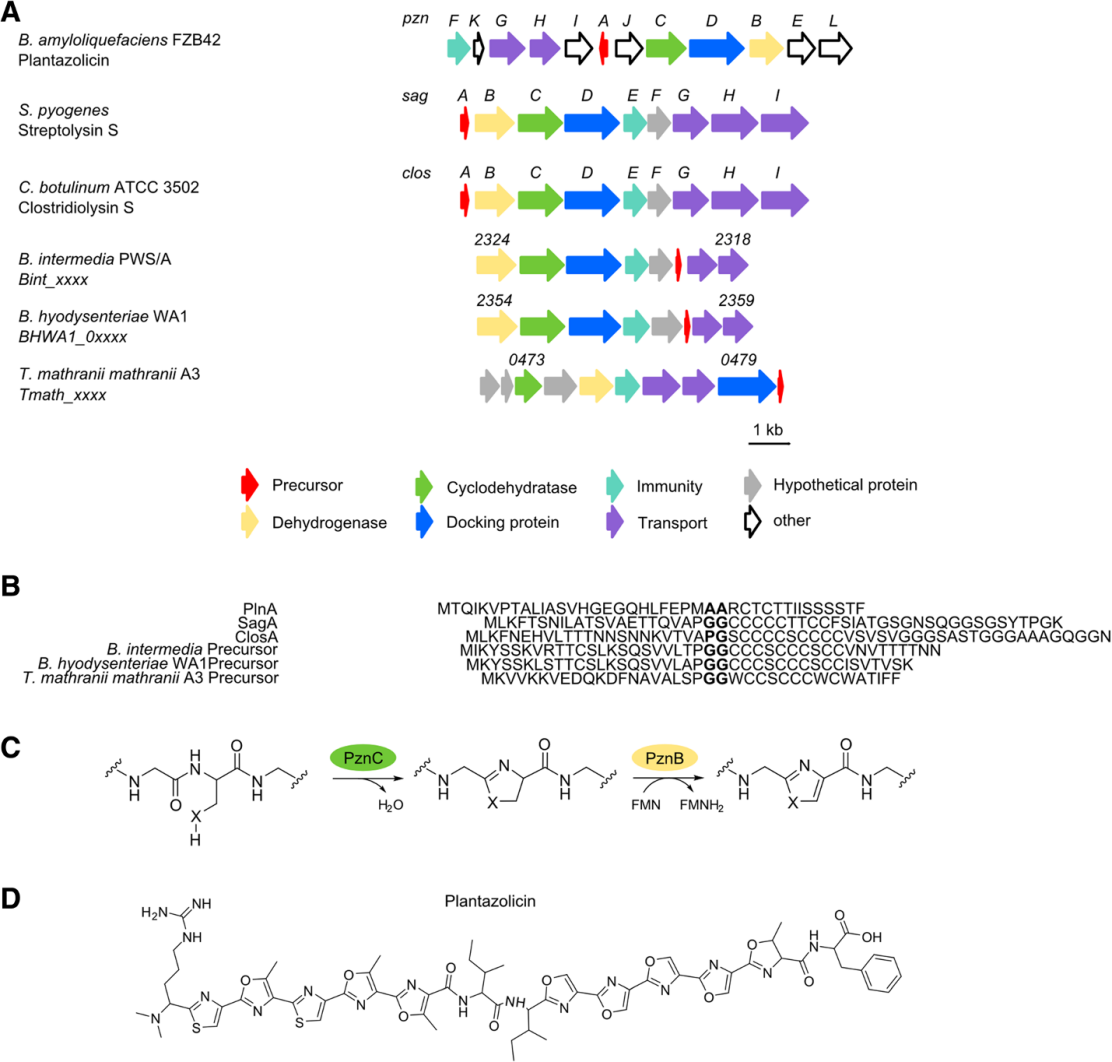
**Detected putative LAP gene cluster. A** Gene cluster of plantazolicin (*pzn*) (*B. amyloliquefeaciens* FZB42), streptolysin S (*sag*) (*S. pyrogenes*) and clostridiolysin S (*clos*) (*C. botulinum* ATCC 3502) in comparison to putative LAP gene clusters of *B. intermedia*, *B. hyodysenteriae* and *T. mathranii mathranii* A3; Numbers represent the locus tag for each gene within the genome sequence of each organism. **B** Comparison of precursor peptides of plantazolicin (PlnA), streptolysin S (SagA), clostridiolysin S (ClosA) with putative precursor peptides of *B. intermedia*, *B. hyodysenteriae*, and *T. mathranii mathranii* A3; Cleavage site of leader and core peptide in bold. **C** Introduction of heterocycles in plantazolicin by cyclodehydrogenase (PznC) and dehydrogenase (PznB) enzyme complex, X = S,O. **D** Chemical structure of plantazolicin.

Many RiPPs are characterized by the presence of heterocyclic functional groups, such as oxazoles and thiazoles. One such group are the linear azol(in)e-containing peptides (LAP), whose heterocycles are derived from the cysteine, serine and threonine of a small precursor peptide [1]. LAP comprise of four essential components: a precursor peptide (known as ‘A’), and a heterotrimeric enzyme complex consisting of a dehydrogenase (‘B’) and cyclodehydratase (‘C’ and ‘D’). Biosynthetically, the first step towards a LAP is the formation of an azoline-heterocycle by the ‘C/D’ complex from serine or threonine and a cysteine residue, followed by dehydrogenation by ‘B’ leading to the corresponding azole (Figure 6C).

Known LAP include streptolysin S (*Streptococcus pyogenes*) [44], microcin B17 (*Escherichia coli*) [45], plantazolicin (*Bacillus amyloliquefaciens* FBZ42) [46, 47] (Figure 6D), goadsporin (*Streptomyces* sp. TP- A0584) [48, 49] and clostridiolysin S (*Clostridium botulinum*) [50]. Despite the fact that the ‘BCD’ enzyme complex exhibits rather low amino acid identity between LAP loci, several studies have shown that ‘BCD’ genes from one LAP biosynthetic gene cluster can complement different LAP synthesis pathways, with the precursor peptide being converted into the active RiPP [47, 51]. As a result, these genes can be used for genome mining approaches [24].

The detected LAP gene clusters are found exclusively in the phyla of Firmicutes and Spirochaetes (Table 5). The gene cluster for clostridiolysin S is conserved in almost all *Clostridium botulinum* strains [50], except the strains BKT015925 and E3 str. Alaska E43, where it is absent. Like other LAP, the complete structure of clostridiolysin S has not yet been solved, owing to the difficulty inherent in the structure elucidation of heterocycles [50]. Several strains within the genus *Brachyspira* (*B. pilosicoli* 95/1000, *B. intermedia* PWS/A, *B. murdochii* 56*–*150 and *B. hyodysenteriae* WA1) also share an identical gene cluster, with only the precursor peptide of *B. hyodysenteriae* WA1 having a slightly different amino acid sequence (Figure 6A & B). The LAP gene cluster contained with the genome of *Thermoanaerobacter mathranii mathranii* A3 has a different gene organization.

**Table 5:** **Detected LAP gene cluster**

	Phylum	Locus Tag of dehydrogenase	Similar to ^†^	Reference ^#^
*Clostridium botulinum* A2 BoNT/A2 Kyoto-F	Firmicutes	*CLM_0573*	clostridiolysin S	
*Clostridium botulinum* A BoNT/A1 ATCC 19397	Firmicutes	*CLB_0528*	clostridiolysin S	
*Clostridium botulinum* A BoNT/A1 Hall	Firmicutes	*CLC_0561*	clostridiolysin S	
*Clostridium botulinum* BoNT/B1 Okra	Firmicutes	*CLD_0261*	clostridiolysin S	
*Clostridium botulinum* BoNT/A3 Loch Maree	Firmicutes	*CLK_3698*	clostridiolysin S	
*Clostridium botulinum* Ba4 str. 657	Firmicutes	*CLJ_B0564*	clostridiolysin S	
*Clostridium botulinum* F230613	Firmicutes	*CBF_0535*	clostridiolysin S	
*Clostridium botulinum* H04402 065	Firmicutes	*H04402_00508*	clostridiolysin S	
*Clostridium botulinum* A ATCC 3502	Firmicutes	*CBO0487*	clostridiolysin S	[50]
*Clostridiumbotulinum* F str. Langeland	Firmicutes	*CLI_0567*	clostridiolysin S	
*Thermoanaerobacter mathranii mathranii* A3, DSM 11426	Firmicutes	*Tmath_0475*	**	
*Brachyspira hyodysenteriae* WA1	Spirochaetes	*BHWA1_02354*		
*Brachyspira pilosicoli* 95/1000	Spirochaetes	*BP951000_0919*	*	
*Brachyspira intermedia* PWS/A	Spirochaetes	*Bint_2324*	*	
*Brachyspira murdochii* 56*–*150, DSM 12563	Spirochaetes	*Bmur_0997*	*	

*All strains share an identical precursor peptide sequence; **no precursor peptide could be annotated; ^†^Cluster shows similarities to characterized RiPP cluster; ^#^Cluster was previously detected by genome mining approaches.

### Thiopeptides

**Figure 7:**
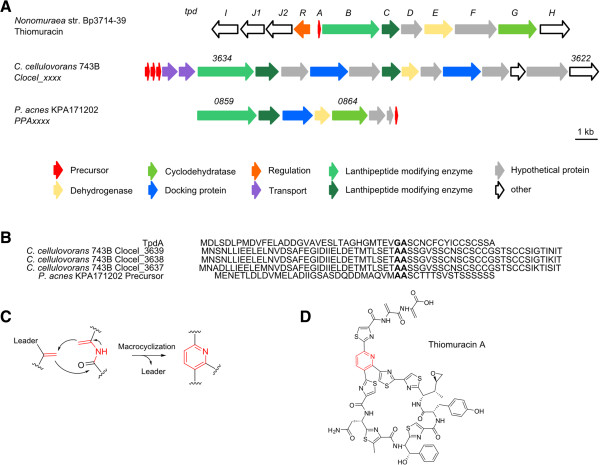
**Detected putative thiopeptides. A** Gene cluster of thiomuracin (*tpd*) (*Nonomuraea* str. Bp3714-39) in comparison to putative thiopeptide gene clusters of *C. cellulovorans* 743B and *P. acnes* KPA171202; Numbers represent the locus tag for each gene within the genome sequence of each organism. **B** Comparison of precursor peptides of thiomuracin (TpdA) and putative precursor peptides of *C. cellulovorans* 753B and *P. acnes* KPA171202; Cleavage site of leader and core peptide in bold. **C** Introduction of the central nitrogen heterocycle (red) in series d thiopeptides. **D** Chemical structure of thiomuracin A.

Thiopeptides are characterized by a highly modified peptide macrocycle including several thiozole rings, a six-membered nitrogenous ring (either present as piperidine, dehydropiperidine or pyridine) and a side chain containing multiple dehydrated amino acid residues [1, 52, 53]. The introduction of a second macrocycle increases the complexity of these peptides and tryptophan-derived quinaldic acid or indolic acid residues are incorporated into the peptide scaffold. As for LAP biosynthesis, the thiozole rings are formed by dehydrogenation and cyclodehydratation of serine and cysteine residues [1, 52, 53]. The central nitrogen heterocycle is installed by a cycloaddition of two dehydroalanines catalyzed by similar proteins found in lanthipeptide biosynthesis (Figure 7C). Depending on the oxidation state and substitution pattern of the central nitrogen heterocycle, thiopetides are classified into different series (A-E) [1, 52, 53]. Thiomuracin A, isolated from a *Nonomuraea* species with strong activity against *S. aureus*[54] (Figure 7D), represents a series D thiopeptide with a tri-substituted pyridine ring as the central nitrogen heterocycle (Figure 7C). Besides the strong activity of many thiopeptides against Gram-positive bacteria by interfering with protein synthesis, some show additional antimalarial or anticancer activities (thiostrepton A) [1, 52, 53].

Two putative thiopeptide gene clusters have been detected in *C. cellulovorans* 743B and *P. acnes* KPA171202, both most likely encoding a series D thiopeptide (Table 6). The *C. cellulovorans* gene cluster (Figure 7A) encodes a LAP-like portion, with genes encoding the dehydrogenase and docking protein of a potential LAP but missing the cyclodehydratase protein. Furthermore lanthionine modifying proteins and three putative precursor peptides are located within the cluster, differing slightly in their protein sequence and showing greater similarity to LAP than to lanthipeptide precursors (Figure 7B).

**Table 6:** **Detected thiopeptide gene cluster**

	Phylum	Locus Tag of modifying enzyme Lan
*Clostridium cellulovorans* 743B	Firmicutes	*Clocel_3634*
*Propionibacterium acnes* KPA171202	Actinobacteria	*PPA0859*

### Nitrile hydratase-related leader peptides (NHLP)

An intersection between LAP and lanthipeptides is formed by the class of NHLP (nitrile hydratase-related leader peptides) and Niff11 (nitrogen-fixing) related RiPPs [55]. On the one hand representatives of these RiPPs can contain the cyclodehydratase and dehydrogenase enzyme complex of LAP (introduction of heterocycles), and on the other hand the LanM enzymes involved in lanthipeptide biosynthesis (see above) [55]. A characteristic feature of these RiPPs are their precursor peptides, where NHLP precursors show sequence similarity with the α-subunit of nitrile hydratases (NHase), but without the active site motif [55]. The so-called Niff11 precursor peptides resemble an uncharacterized protein, which can be frequently found in nitrogen-fixing bacteria (including cyanobacteria) [55]. Compared to LAP, whose typical leader peptide sequences are about 24 amino acids in length, NHLP/Niff11 precursor peptides have much longer leader sequences, typically in the range of 70–83 amino acids [55]. The leader sequence is often terminated by a glycine-glycine motif. In contrast to their *N*-terminal sequences, the *C*-terminal ends of NHLP/Niff11 precursors vary considerably between different gene clusters and are rich in cysteine, serine and threonine, which are required for the posttranslational modifications [55].

**Figure 8:**
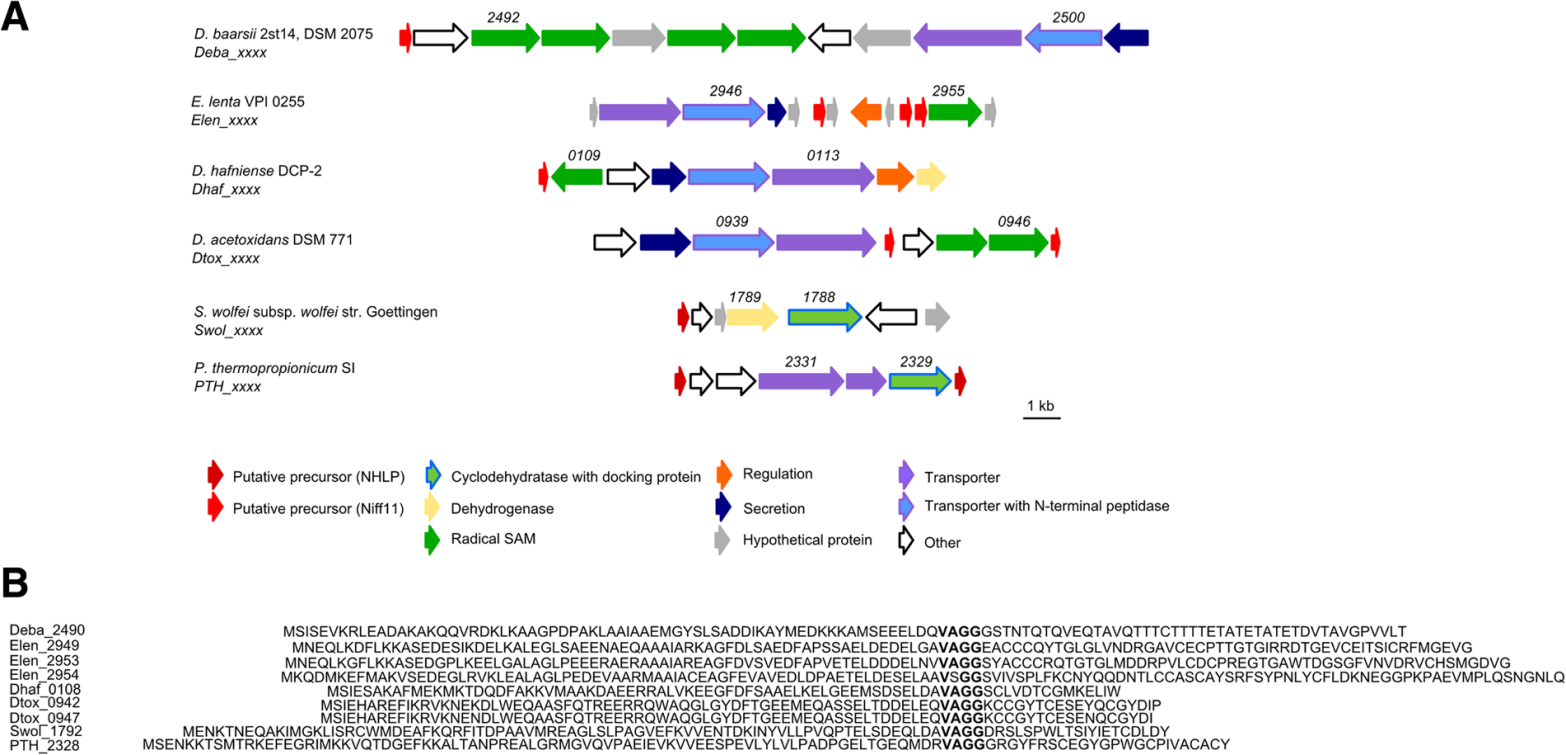
**Detected putative NHLP/** **Niff. A** Structure of putative NHLP/Niff related gene clusters of *D. baarsii* 2st14, *E. lenta* VPI 0255, *D. hafniense* DCP-2, *D. acetoxidans* DSM 771, *S. wolfei* subsp. *wolfei* str. Goettingen, *P. thermopropionicum* SI; Numbers represent the locus tag for each gene within the genome sequence of each organism. **B** Comparison of the putative precursor peptides with VAGG-motif separating the leader and core peptide in bold.

The putative anaerobic NHLP/Niff11 clusters are located exclusively in the Actinobacteria, δ-Proteobacteria and Firmicutes phyla and all putative precursor peptides are annotated as NHLP or Niff11-superfamily proteins. The leader sequences (taken as the amino acid sequence before the GG motif) have a range between 66–85 amino acids, whilst the core sequences, taken as the amino acid sequence following the conserved VAGG or VSGG motif, are quite variable in length (14–59 amino acids) (Table 7, Figure 8B). The number of putative precursor peptides also differs from one to three depending on the individual gene cluster (Figure 8A). It is striking that cyclodehydratase and dehydrogenase related genes were only observed in the gene clusters present in *Syntrophomonas wolfei* subsp. *wolfei* str. Goettingen and *Pelotomaculum thermopropionicum* (Figure 8A). In the other cases a transporter with an *N*-terminal peptidase was identified, as well as several radical SAM proteins, which may be responsible for the modification steps of the NHLP/Niff11 precursors. Furthermore, proteins important for secretion are also located within several of the gene clusters (Figure 8A).

**Table 7:** **Detected putative NHLP**/**Niff11**-**like gene cluster**

	Phylum	Precursor (Leader:Core)^+^	Gene tag of precursor peptides	Reference ^#^
*Eggerthellalenta* VPI 0255	Actinobacteria	121 (71:50)	*Elen_2949*	
		122 (71:51)	*Elen_2953*	
		130 (71:59)	*Elen_2954*	
*Desulfarculusbaarsii* 2st14, DSM 2075	δ- Proteobacteria	111 (71:40)	*Deba_2490*	
*Syntrophomonas wolfei* subsp. *wolfei* str. Goettingen	Firmicutes	102 (82:20)	*Swol_1792*	[55]
*Desulfotomaculum acetoxidans* DSM 771	Firmicutes	84 (66:18)	*Dtox_0942*	
		83 (66:17)	*Dtox_0947*	
*Desulfitobacterium hafniense* DCP-2	Firmicutes	80 (66:14)*	*Dhaf_0108*	
*Desulfitobacterium hafniense* Y51	Firmicutes	80 (66:14)*	*DSY0169*	
*Pelotomaculum thermopropionicum* SI	Firmicutes	109 (85:24)	*PTH_2328*	[55]
		102 (85:17)	*PTH_2334*	

^+^amino acid length of precursor sequence (length of leader peptide : core peptide); *identical sequences; ^#^Cluster was previously detected by genome mining approaches.

### Lasso peptides

**Figure 9:**
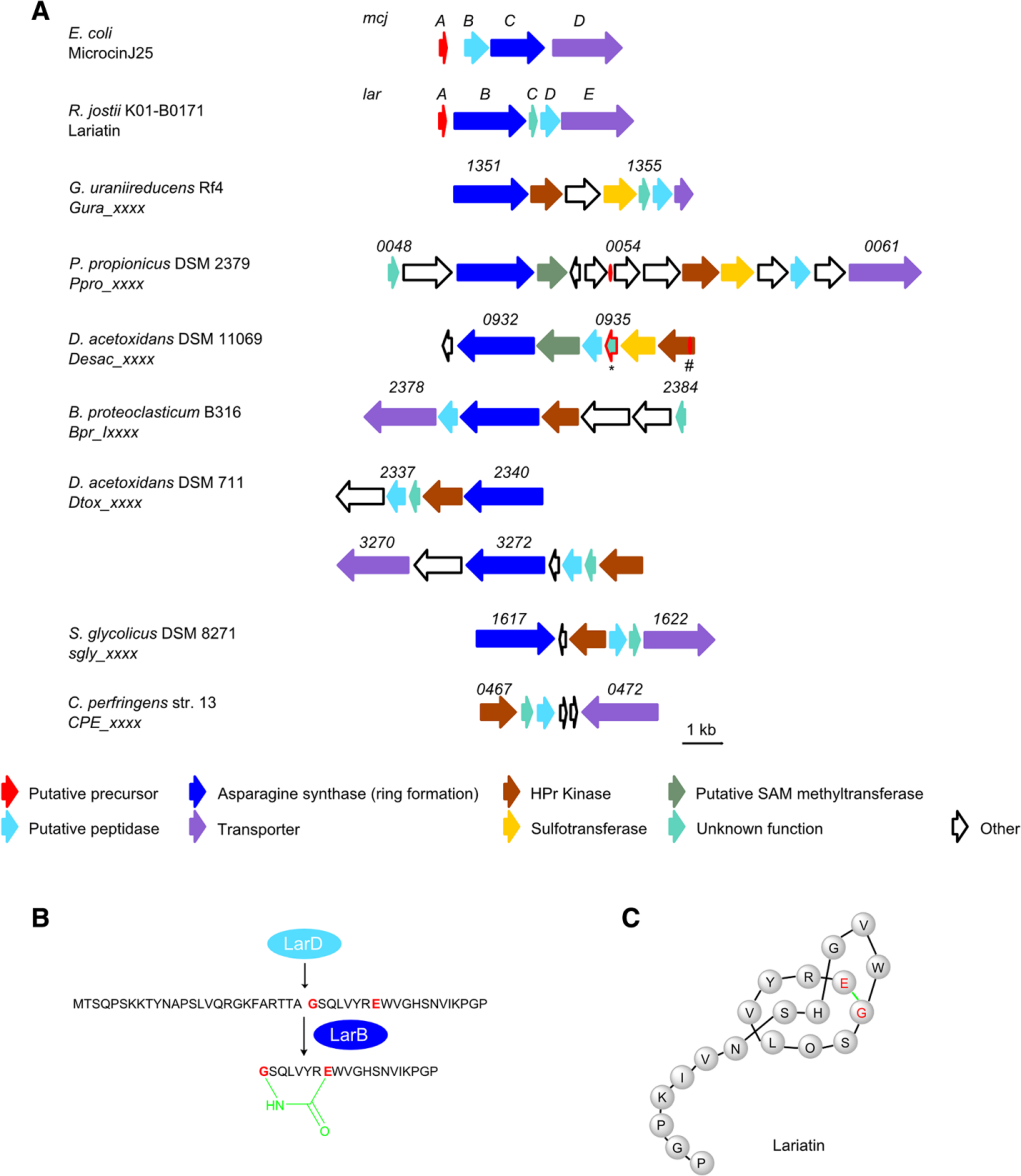
**Detected putative lasso peptides. A** Microcin J25 (*mcj*) (*E. coli*) and Lariatin (*lar*) (*R. jostii* K01-B0171) gene clusters in comparision to putative lasso peptide gene clusters of *G. uraniireducens* Rf4, *P. propionicus* DSM 2379, *D. acetoxidans* DSM 11069 (* = precursor peptide identified in this study, # = precursor peptide identified by [71]), *B. proteoclasticum* B313, *D. acetoxidans* DSM 771, *S. glycolicus* DSM 8271 and *C. perfringens* str. 13; Annotation of the putative precursor peptide was not conclusively possible in most cases; Numbers represent the locus tag for each gene within the genome sequence of each organism. **B** Cleavage of the lariatin precursor peptide by a putative protease (LarD); Isopeptide bond (green) formation by LarB between the *N*-terminal amino acid glycine (red) and a glutamate (red) leads to the formation of a 8- membered macrolactame ring in lariatin. **C** Lasso peptide structure of lariatin (isopeptide bond (green)).

Lasso peptides are among the most extraordinary RiPPs, and their rigid structure gives them enormous stability against heat, chemical attack and proteases [1, 56, 57]. So named because of their particular knotted structure, the lasso peptides are usually 16–23 amino acids in length and contain an 8–9 membered macrolactam ring, which is formed between the *N*-terminal amino group and the carboxylate of a conserved aspartate or glutamate residue at position 8 or 9, by a putative asparagine synthase like enzyme, resulting in a *C*-terminal loop and tail formation [1, 56, 57] (Figure 9B & C). Three subgroups of the lasso peptides have been characterized. The prototypical members of the group I lasso peptides include siamycin I [58], siamycin II [58] and RP71955 [59], all of which possess two disulfide bonds and an *N*-terminal cysteine [1, 56, 57]. In contrast, group II lasso peptides contain no disulfide bonds, and the *N*-terminal amino acid is glycine [1, 56, 57], with examples in the form of microcin J25 [60, 61], lariatin [62] and capistruin [63, 64]. Lasso peptide BI-32169 [65, 66] is the only member of group III, having one disulfide bridge and glycine as the *N*-terminal amino acid [1, 56, 57].

Studies on the biosynthesis of microcin J25 from *E. coli* AY25 [67, 68] and capistruin from *Burkholderia thailandensis*[63, 64] have shown that four genes (‘A-D’) are necessary for lasso peptide formation. In each case, the leader sequence is cleaved by an ATP-dependent protease (‘B’) from the precursor peptide (‘A’), with the simultaneous activation of the aspartate or glutamate residues [1, 56, 57]. Isopeptide bond formation is catalyzed by an ATP-dependent enzyme (‘C’), which has similarities to asparagine synthetase B, and the resulting product is transported out of the cell through ‘D’, which also ensures immunity of the producer to the mature RiPP [1, 56, 57]. Only the first eight *N*-terminal amino acids and the second last threonine of the leader sequence are required for its recognition by the modifying enzymes [69]. Due to conservation of the ‘B’ and ‘C’ enzymes, as well as conserved motifs in the precursor sequences, these can all be used as the basis for genome mining [24, 56, 67, 70–72].

Previous attempts at genome mining for lasso peptides identified putative gene clusters within the following anaerobe genomes: *Spirochaeta smaragdinae* DSM 11293, *Syntrophomonas wolfei* subsp. *wolfei* str. Goettingen, *Treponema pallidum*, *Treponema cuniculi paraluiscuniculi* A*, Pelobacter propionicus* DSM 2379, *Desulfobacca acetoxidans* DSM 111069 and *Geobacter uraniireducens*[71, 72]. However, upon closer investigation, several of these gene clusters were either undetected in the present study, or lacked the necessary genes encoding the characteristic lasso peptide modifying enzymes and as such they were not included in the current analysis. In the case of *Desulfobacca acetoxidans* both studies identified identical gene clusters for putative lasso peptides, with the only difference being the prediction of the precursor peptide (Figure 9A (* = precursor peptide identified in this study, # = precursor peptide identified by [71])).

The biosynthetic gene clusters for microcin J25 and lariatin are shown in Figure 9A [73, 74]. Unlike microcin J25 and other lasso peptides, lariatins A and B, produced by *Rhodococcus jostii*, are formed by a five-gene cluster, *larABCDE*. Similar to other lasso peptides, LarA is the precursor peptide which is processed by LarB, LarC and LarD and then exported by the transporter LarF [73]. Whilst LarB and LarD appear to have similar functions, the role of LarC remains unclear, although it appears that *larC* is specific for Gram-positive bacteria [73]. Indeed, this appeared to be the case, as all anaerobic strains in which lasso peptide gene clusters were identified (Table 8) were Gram-positive and contained *larC* orthologues (Figure 9A). Interestingly, additional enzymes, such as a HPr kinase and a sulfotransferase, were also identified in some gene clusters. The role that these proteins play in the modification of the precursor peptides is currently unclear, although they may be involved in previously unidentified lasso peptide modifications [55].

**Table 8:** **Detected putative lasso peptides**

	Phylum	Gene tag of asparagine synthase	Reference ^#^
*Geobacter uraniireducens* Rf4	δ- Proteobacteria	*Gura_1351*	[71, 72]
*Pelobacter propionicus* DSM 2379	δ- Proteobacteria	*Ppro_0050*	[71]
*Desulfobacca acetoxidans* ASRB2, DSM 111069	δ- Proteobacteria	*Desac_0932*	[71, 72]
*Butyrivibrio proteoclasticum* B316	Firmicutes	*bpr_I2380*	
*Desulfotomaculum acetoxidans* DSM 771	Firmicutes	*Dtox_2340*	
		*Dtox_3272*	
*Syntrophobotulus glycolicus* FIGlyR, DSM 8271	Firmicutes	*Sgly_1617*	
*Clostridium perfringens* 13	Firmicutes	*CPE0468*	

^#^Cluster was previously detected by genome mining approaches.

### Lactococcin

**Figure 10:**
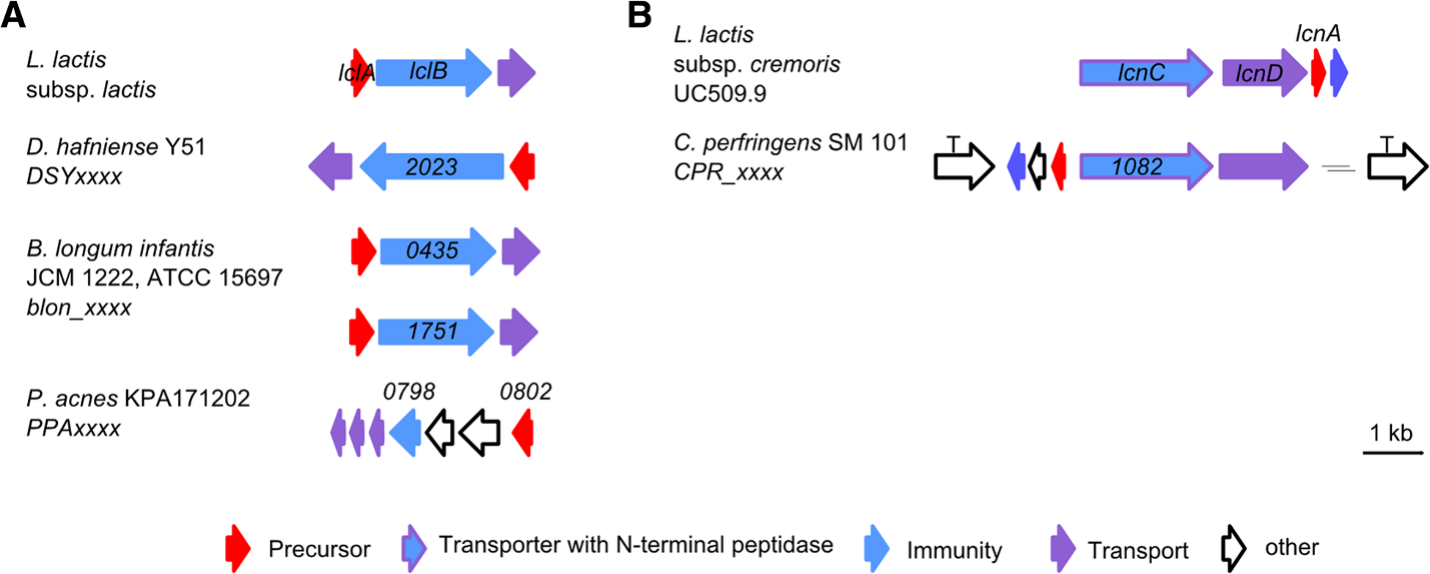
**Detected putative lactococcins like RiPPs. A** Lactococcin 972 gene cluster (*lcl*) of *L. lactis* subsp. *lactis* in comparison to detected putative lactococcin 972 like gene clusters in *D. hafniense* Y51, *B. longuminfantis* JCM 1222 and *P. acnes* KPA171202; Numbers represent the locus tag for each gene within the genome sequence of each organism. **B** Lactococcin A gene cluster (*lcn*) of *L. lactis* subsp. *cremoris* and detected putative lactococcin A-like gene cluster in *C. perfringens* SM 101; (T = transposase); Numbers represent the locus tag for each gene within the genome sequence of each organism.

Like many other RiPPs, lactococcins possess an *N*-terminal leader sequence, which terminates in a glycine-glycine motif. This motif is an important signal unit for the respective transporters which secrete the substance and simultaneously cleave off the leader sequence [75]. Lactococcin 972 is homodimeric RiPP, which is only encoded by one structural gene [76]. This gene encodes a 91 amino acid precursor peptide of which 25 amino acids comprise the leader sequence and the remainder, the core sequence [76, 77]. In addition to the precursor peptide named LclA the gene cluster encodes a transporter (LclB) and an additional protein that is important for immunity (Figure 10A). Lactococcin 972 blocks the incorporation of lipid II, an essential cell wall building block [77–79].

In almost all propionibacteria a lactococcin 972 like precursor peptide is present, with the exception of *Propionibacterium acnes* ATCC 11828, where it is absent (Figure 10A). The *N*-terminal leader sequence of *P. acnes* 266 includes an additional 23 amino acids in comparison to the other *P. acnes* strains. The gene organization in the *P. acnes* strains is different to *Desulfitobacterium hafniese Y51*, *Bifidobacterium longum infantis* and in comparison to the characterized lactococcin 972 gene cluster of *Lactococcus lactis* (Figure 10A).

Lactococcin like genes were detected in 11 genomes, in particular genes encoding lactococcin 972 and lactococcin A like proteins (Table 9). Unlike lactococcin 972, lactococcin A (*Lactococcus lactis* subsp. *cremoris*, *Lactococcus lactis* subsp. *lactis* biovar *diacetylactis* WM4) is a linear RiPP with a 75 amino acid precursor, containing a 21 amino acid leader sequence [80, 81]. Both lactococcins have a limited spectrum of activity against various *Lactococcus* strains and the antimicrobial effect of these peptides is based on the binding of the peptide core to a mannose-phosphotransferase that is localized in the cell wall of the target organism, resulting in increased cell wall permeability [77, 80, 82]. In lactococcin A biosynthesis, the precursor peptide LcnA is processed by LcnC, a transporter with *N*-terminal peptidase, and secreted by LcnD [81] (Figure 10B). A co-localized self-resistance gene guarantees immunity to lactococcin A. A homologous gene cluster to lactococcin A could be detected in *C. perfringens* SM 101, where the cluster is flanked by transposases (Figure 10B).

**Table 9:** **Detected putative lactococcin**-**like RiPPs**

	Phylum	AA ^+^	Gene tag of precursor	Protein family
*Lactococcuslactis* subsp. lactis	Firmicutes	91	*lclA*	Lactococcin 972 producer
*Bifidobacterium longum infantis* JCM 1222, ATCC 15697	Actinobacteria	135	*Blon_0434*	Lactococcin 972
		148	*Blon_1750*	Lactococcin 972
*Propionibacterium acnes* KPA171202	Actinobacteria	93	PPA_0802*	Lactococcin 972
*Propionibacterium acnes* TypeIA2 P.acn17	Actinobacteria	93	TIA2EST22_04040*	Lactococcin 972
*Propionibacterium acnes* TypeIA2 P.acn31	Actinobacteria	93	TIA2EST36_04010*	Lactococcin 972
*Propionibacterium acnes* TypeIA2 P.acn33	Actinobacteria	93	TIA2EST2_03960*	Lactococcin 972
*Propionibacterium acnes* 266	Actinobacteria	114	PAZ_c08490*	Lactococcin 972
*Propionibacterium acnes* 6609	Actinobacteria	93	TIB1ST10_04145*	Lactococcin 972
*Desulfitobacterium hafniense* Y51	Firmicutes	126	*DSY2024*	Lactococcin 972
*Lactococcus lactis* subsp. *cremoris*	Firmicutes	75	*uc509_p7031*	Lactococcin A producer
*Clostridium perfringens* SM101	Firmicutes	69	*CPR_1081*	Lactococcin A

*Identical precursor sequences; ^+^Number of amino acids encoded by precursor gene.

### Head-to-tail cyclized peptides

As the name head-to-tail (HtT) suggests, these peptides are cyclized between their *N*- and *C*-terminus and range in size between 30–70 amino acids [1]. As with other cyclized peptides, they show a higher stability to heat, pH changes and proteases. These peptides tend to be hydrophobic and exert their effects by the formation of pores in the cell membrane of target organisms [1, 83]. The most famous and best known representative of this group is enterocin AS-48 (*Enterococcus* sp.) [84]. Other HtT-like cyclized peptides are the cyanobactins, amatoxins and cyclotides but they differ in their size and their biosynthetic origin [1, 83]. For example, HtT-cyclized peptides have no additional amino acids at the *C*-terminal end, which contribute to the cyclization and ring formation [1, 83]. It is still not completely clear how the *C*-terminal carboxyl group is activated, however, a protein containing a conserved domain of unknown function is present in most of the identified gene clusters [1, 83]. ATP-binding proteins are also present in the majority of the known gene clusters, which may also be involved in the activation of the carboxyl group [1, 83]. Because of their presence in many HtT-cyclized protein gene clusters these genes can be used for genome mining approaches.

**Figure 11:**
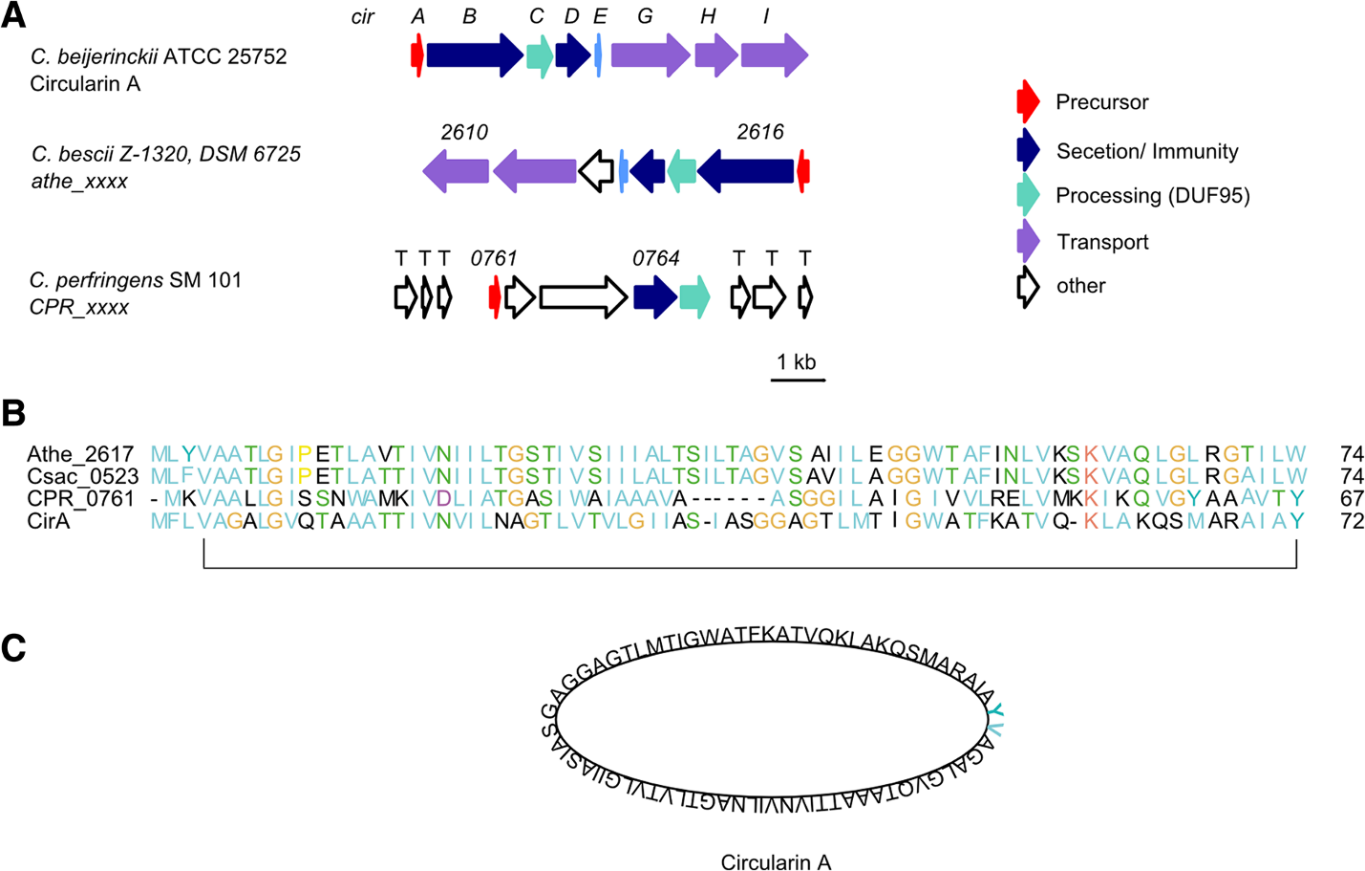
**Detected circularin A**-**like RiPPs. A** Circularin A gene cluster (*cir*) of *C. beijerinckii* ATCC 25752 in comparison to putative circularin A like gene cluster of *C. bescii* Z-1320 and *C. perfringens* SM 101; Numbers represent the locus tag for each gene within the genome sequence of each organism. **B** Alignment of circularin A precursor sequence (CirA) and circularin A-like precursor sequences of *C. bescii* Z-1320 (Athe_2617), C. *saccharolyticus* DSM 8903 (Csac_0526) and *C. perfringens* SM 101 (CPR_0761) **C** Amino acid structure of circularin A.

Relatively few HtT-cyclized peptides were identified amongst the genomes analyzed here. Those that were identified were found in the phyla Firmicutes and Chloroflexi (Table 10), with several exhibiting homology to circularin A (Figure 11A), a previously characterized peptide of *Clostridium beijerinckii* ATCC 25752 [85, 86] (Figure 11C). The gene order in the *Caldicellulosiruptor* gene clusters is identical to each other and the precursor sequences differ by only a few amino acids (Figure 11B). The putative circularin A gene cluster of *C. perfringens* SM 101 is quite different, however, and it has limited conservation with the circularin A gene cluster in *C. beijerinckii* ATCC 25752 and is flanked by numerous transposases (Figure 11A).

**Table 10:** **Detected putative head**-**to**-**tail cyclized peptides**

	Phylum	Gene tag of processing enzyme (DUF95)	Similar to ^†^
*Dehalogenimonas lykanthroporepellens* BL-DC-9	Chloroflexi	*Dehly_0974*	
*Chloroflexus aurantiacus* J-10-fl	Chloroflexi	*Caur_1589*	
*Eubacterium limosum* KIST612	Firmicutes	*ELI_3775*	
*Caldicellulosiruptor bescii* Z-1320, DSM 6725	Firmicutes	Athe_2615*	circularin A
*Caldicellulosiruptor saccharolyticus*, DSM 8903	Firmicutes	Csac_0523*	circularin A
		*Csac_2575*	
*Caldicellulosiruptor obsidiansis* OB47	Firmicutes	*COB47_0044*	
*Clostridium perfringens* SM101	Firmicutes	*CPR_0765*	circularin A

*Identical cluster but different putative precursor peptide; ^†^Cluster shows similarities to characterized RiPP cluster.

## Conclusion

Here we have surveyed the genomes of 211 anaerobic bacteria for the presence of RiPP biosynthetic gene clusters. As such, we have identified >25% of anaerobes are capable of producing RiPPs either alone or in conjunction with other secondary metabolites, such as polyketides or non-ribosomal peptides. As with the possession of NRPS and PKS gene clusters, the most likely RiPP producer organisms lie within the phyla Proteobacteria and Firmicutes. However, in contrast to their NRPS and PKS biosynthetic potential, which was minimal, anaerobic Actinobacteria appear to have a greater propensity for RiPP production. Interestingly, we found that the phylum Spirochaetes also contains a number of potential RiPP producing organisms, something that has not previously been found. In general, it also appears that non-pathogenic organisms have a greater potential for RiPP production, which aligns well with what is known about NRPS/PKS potential in anaerobes. Remarkably, anaerobes were found to have the potential to produce a variety of different RiPP classes, with the LAPs and lactococcins appearing to be favored by pathogenic anaerobes, whilst the other classes are more prominent in non-pathogenic isolates. Surprisingly, isolates from extreme environments contain a wide range of different RiPPs, in particular head-to-tail cyclized peptides and lanthipeptides. Despite the fact that their environmental niche is already restricted, it appears as though it must still be necessary for these organisms to have some way to defend themselves against competitors. In total we identified 81 putative RiPP clusters of which 43 had not been previously described and appear to be unique among known RiPP biosyntetic gene clusters. Furthermore, we were able to identify 23 gene clusters with similarities to known RiPP biosynthetic gene clusters, but that have not been previously identified in anaerobes and we were able to confirm a further 15 previously identified RiPP gene clusters.

Amongst the analyzed genomes, several gene clusters with good correlation to known RiPPs were identified. These include a number of potential class II lanthipeptides from the phyla Firmicutes and Actinobacteria, with similarity to the lichenicidin gene cluster from *Bacillus licheniforme*; sactipeptides identified in the phylum Firmicutes with similarities to the thuricin CD gene cluster of *B. thuringiensis*; head-to-tail cyclized peptides within the phyla Chloroflexi and Firmicutes with homology to the circularin A biosynthetic gene cluster from *C. beijerinckii* ATCC 25752; and lactococcin 972-like RiPPs from the phylum of Actinobacteria. The distribution of similar gene clusters amongst diverse organisms suggests that horizontal gene transfer has been active in the distribution of RiPP gene clusters amongst organisms that share similar environments.

Despite the fact that several identified gene clusters and precursor peptides show similarities to previously characterized RiPPs, in many instances the prediction of the final products remains difficult. Differences in the precursor peptide sequence between similar RiPP products may have an impact on the final modified structure of the peptide, meaning that prediction of RiPP homology between species where a similar gene cluster exists is also difficult.

In consideration of the increasing number of multiresistant strains, RiPPs are a promising alternative to classical antibiotic treatment. This investigation is the first report of the potential of anaerobic bacteria for the production of RiPPs and the detected putative RiPPs may represent future lead compounds in the fight against multirestistant pathogens. Nevertheless, the identification of all these potential metabolites remains a challenge for the future and more methods are needed to connect the detected genotypes to chemotypes [87].

## Methods

### Genome sequences

Complete and published genome sequences of 211 anaerobic bacteria (Additional file 1: Table S1) were obtained from the NCBI Refseq and draft genome repository.

### Analysis of anaerobe genomes

Genomes were analyzed for the presence of RiPP encoding gene clusters by using the web-based bioinformatic tools antiSMASH [15, 16], Bagel and bactibase [17, 18]. Predicted gene clusters from each of the database outputs were inspected manually and compared using BLAST searches. Putative gene clusters were classified according to Arnison et al. [1] (antiSMASH data collected in April/ May 2012; Bagel database data collected in January 2014).

## Electronic supplementary material

Additional file 1: Table S1: Genomes (finished and published) of anaerobic bacteria analyzed in this study. (DOCX 23 KB)
