# Supplementary material for: Genome mining for ribosomally synthesized and post-translationally modified peptides (RiPPs) in anaerobic bacteria
Source: BMC Genomics. 2014 Nov 18;15(1):983. doi: 10.1186/1471-2164-15-983 (PMC4289311; doi:10.1186/1471-2164-15-983)
Supplement: Supplementary file 1 — Additional file 1: Table S1: Genomes (finished and published) of anaerobic bacteria analyzed in this study. (DOCX 23 KB) [file 12864_2014_6858_MOESM1_ESM.docx]

**Table S1: Genomes (finished and published) of anaerobic bacteria analyzed in this study**

| *Bifidobacterium bifidum* S17 | Actinobacteria |
| --- | --- |
| *Bifidobacterium bifidum* PRL2010 | Actinobacteria |
| *Bifidobacterium longum infantis* 157F-NC | Actinobacteria |
| *Bifidobacterium longum infantis* JCM 1217 | Actinobacteria |
| *Bifidobacterium longum infantis* ATCC 15697 = *Bifidobacterium longum infantis* JCM 1222 | Actinobacteria |
| *Bifidobacterium breve* UCC2003 | Actinobacteria |
| *Bifidobacterium animalis lactis* CNCM-I-2494 | Actinobacteria |
| *Bifidobacterium longum* NCC2705 | Actinobacteria |
| *Bifidobacterium longum* DJO10A | Actinobacteria |
| *Bifidobacterium longum susp. longum* JDM301 | Actinobacteria |
| *Bifidobacterium longum susp. longum* BBMN68 | Actinobacteria |
| *Bifidobacterium longum susp. longum* KACC 91563 | Actinobacteria |
| *Bifidobacterium animalis lactis* AD011 | Actinobacteria |
| *Bifidobacterium animalis lactis* Bl-04 | Actinobacteria |
| *Bifidobacterium animalis lactis* DSM 10140 | Actinobacteria |
| *Bifidobacterium dentium* Bd1 | Actinobacteria |
| *Bifidobacterium animalis lactis* BB-12 | Actinobacteria |
| *Bifidobacterium animalis lactis* V9 | Actinobacteria |
| *Bifidobacterium animalis lactis* BLC1 | Actinobacteria |
| *Bifidobacterium animalis subsp. animalis* ATCC 25527 | Actinobacteria |
| *Propionibacterium acnes* KPA171202 | Actinobacteria |
| *Propionibacterium freudenreichii shermanii* CIRM-BIA1 | Actinobacteria |
| *Propionibacterium acnes* TypeIA2 P.acn17 | Actinobacteria |
| *Propionibacterium acnes* TypeIA2 P.acn31 | Actinobacteria |
| *Propionibacterium acnes* TypeIA2 P.acn33 | Actinobacteria |
| *Propionibacterium acnes* 266 | Actinobacteria |
| *Propionibacterium acnes* 6609 | Actinobacteria |
| *Propionibacterium acnes* ATCC 11828 | Actinobacteria |
| *Slackia heliotrinireducens* RHS 1, DSM 20476 | Actinobacteria |
| *Eggerthella lenta* VPI 0255 | Actinobacteria |
| *Cryptobacterium curtum* 12-3, DSM 15641 | Actinobacteria |
| *Atopobium parvulum* IPP 1246, DSM 20469 | Actinobacteria |
| *Olsenella uli* VPI, DSM 7084 | Actinobacteria |
| *Bacteroides fragilis* 630R | Bacteroidetes |
| *Bacteroides thetaiotaomicron* VPI-5482 | Bacteroidetes |
| *Bacteroides fragilis* YCH46 | Bacteroidetes |
| *Bacteroides fragilis* NCTC 9343 | Bacteroidetes |
| *Bacteroides vulgatus* ATCC 8482 | Bacteroidetes |
| *Bacteroides helcogenes* P36-108 | Bacteroidetes |
| *Bacteroides salanitronis* BL78 | Bacteroidetes |
| *Porphyromonas gingivalis* ATCC 33277 | Bacteroidetes |
| *Porphyromonas gingivalis* TDC60 | Bacteroidetes |
| *Porphyromonas gingivalis* W83 | Bacteroidetes |
| *Parabacteroides distasonis* ATCC 8503 | Bacteroidetes |
| *Odoribacter splanchnicus* 1651/6, DSM 220712 | Bacteroidetes |
| *Paludibacter propionicigenes* WB4 | Bacteroidetes |
| *Prevotella ruminicola* 23 | Bacteroidetes |
| *Chlorobium tepidum* TLS | Chlorobi |
| *Dehalococcoides ethenogenes*  195 | Chloroflexi |
| *Dehalococcoides* sp. CBDB1 | Chloroflexi |
| *Dehalococcoides* sp. BAV1 | Chloroflexi |
| *Dehalococcoides* sp. VS | Chloroflexi |
| *Dehalogenimonas lykanthroporepellens* BL-DC-9 | Chloroflexi |
| *Chloroflexus aurantiacus* J-10-fl | Chloroflexi |
| *Desulfurispirillum indicum* S5 | Chrysiogenetes |
| *Hippea maritima* MH2, DSM 10411 | Proteobacteria |
| *Desulfarculus baarsii*  2st14, DSM 2075 | Proteobacteria |
| *Geobacter uraniireducens*  Rf4 | Proteobacteria |
| *Geobacter lovleyi*  SZ | Proteobacteria |
| *Geobacter bemidjiensis*  Bem | Proteobacteria |
| *Geobacter sulfurreducens*  KN400 | Proteobacteria |
| *Geobacter sulfurreducens*  PCA | Proteobacteria |
| *Geobacter metallireducens*  GS-15 | Proteobacteria |
| *Pelobacter carbinolicus* DSM 2380 | Proteobacteria |
| *Pelobacter propionicus* DSM 2379 | Proteobacteria |
| *Synthrophus aciditrophicus* SB | Proteobacteria |
| *Desulfobacca acetoxidans* ASRB2, DSM 111069 | Proteobacteria |
| *Desulfomicrobium baculatum* X, DSM 4028 | Proteobacteria |
| *Desulfovibrio vulgaris* DP4 | Proteobacteria |
| *Desulfovibrio magneticus* RS-1 | Proteobacteria |
| *Desulfovibrio vulgaris* Hildenborough | Proteobacteria |
| *Desulfovibrio alaskensis* G20 | Proteobacteria |
| *Desulfohalobium retnaense* HR100, DSM 5692 | Proteobacteria |
| *Desulfatibacillum alkenivorans* AK-01 | Proteobacteria |
| *Desulfobacterium autotrophicum* HRM2 | Proteobacteria |
| *Desulfobulbus propionicus* 1pr3, DSM 2032 | Proteobacteria |
| *Desulfotalea psychrophila* LSv54 | Proteobacteria |
| *Nautilia profundicola* AmH | Proteobacteria |
| *Dichelobacter nodosus* VCS1703A | Proteobacteria |
| *Deferribacter desulfuricans* SSM1 | Deferribacteres |
| *Calditerrivibrio nitroreducens* Yu37-1, DSM 19672 | Deferribacteres |
| *Denitrovibrio acetiphilus* N2460, DSM 12809 | Deferribacteres |
| *Flexistipes sinusarabici* MAS10, DSM 4947 | Deferribacteres |
| *Thermus scotoductus* SA-01 | Deinococcus-Thermus |
| *Elusimicrobium minutum* Pei 191 | Elusibacteria |
| *Fibrobacter succinogenes* S85 | Fibrobacteres |
| *Clostridium acetobutylicum* DSM 1731 | Firmicutes |
| *Clostridium acetobutylicum* ATCC 824 | Firmicutes |
| *Clostridium acetobutylicum* EA 2018 | Firmicutes |
| *Clostridium beijerinckii* NCIMB 8052 | Firmicutes |
| *Clostridium botulinum* A2 BoNT/A2 Kyoto-F | Firmicutes |
| *Clostridium botulinum* A BoNT/A1 ATCC 19397 | Firmicutes |
| *Clostridium botulinum* A BoNT/A1 Hall | Firmicutes |
| *Clostridium botulinum* BoNT/B1 Okra | Firmicutes |
| *Clostridium botulinum* BoNT/A3 Loch Maree | Firmicutes |
| *Clostridium botulinum* Ba4 str. 657 | Firmicutes |
| *Clostridium botulinum* BKT015925 | Firmicutes |
| *Clostridium botulinum* F230613 | Firmicutes |
| *Clostridium botulinum* H04402 065 | Firmicutes |
| *Clostridium botulinum* A ATCC 3502 | Firmicutes |
| *Clostridium botulinum* F str. Langeland | Firmicutes |
| *Clostridium botulinum* E3 str. Alaska E43 | Firmicutes |
| *Clostridium cellulolyticum* H10 | Firmicutes |
| *Clostridium cellulovorans* 743B, ATCC 35296 | Firmicutes |
| *Clostridium clariflavum* EBR45, DSM 19732 | Firmicutes |
| *Clostridium difficile* 630 | Firmicutes |
| *Clostridium difficile* CD196 | Firmicutes |
| *Clostridium difficile* R20291 | Firmicutes |
| *Clostridium kluyveri* DSM 555 | Firmicutes |
| *Clostridium lentocellum* RHM5, DSM 5427 | Firmicutes |
| *Clostridium ljungdahlii* DSM 13528 | Firmicutes |
| *Clostridium novyi* NT | Firmicutes |
| *Clostridium perfringens* ATCC 13124 | Firmicutes |
| *Clostridium perfringens* SM 101 | Firmicutes |
| *Clostridium perfringens* 13 | Firmicutes |
| *Clostridium tetani* Massachusetts E88 | Firmicutes |
| *Clostridium thermocellum* DSM 1313 | Firmicutes |
| *Clostridium thermocellum* ATCC 27405 | Firmicutes |
| *Clostridium saccharolyticum* WM1, DSM 2544 | Firmicutes |
| *Clostridium sticklandii* DSM 519 | Firmicutes |
| *Clostridium* sp. SY8519 | Firmicutes |
| *Heliobacterium modesticaldum* str. Ice1 | Firmicutes |
| *Finegoldia magna* ATCC29328 | Firmicutes |
| *Anaerococcus prevotii* PC1, DSM 20548 | Firmicutes |
| *Symbiobacterium thermophilum* IAM 14863 | Firmicutes |
| *Eubacterium eligens* ATCC 27750 | Firmicutes |
| *Eubacterium limosum* KIST612 | Firmicutes |
| *Eubacterium rectale* ATCC 33656 | Firmicutes |
| *Syntrophothermus lipocalidus* DSM 12680 | Firmicutes |
| *Syntrophomonas wolfei* subsp. wolfei str. Goettingen | Firmicutes |
| *Butyrivibrio proteoclasticum* B316 | Firmicutes |
| *Ruminococcus albus* 7, ATCC 27210 | Firmicutes |
| *Oscillibacter valericigenes* Sjm18-20 | Firmicutes |
| *Desulfotomaculum reducens* MI-1 | Firmicutes |
| *Desulfotomaculum acetoxidans* DSM 771 | Firmicutes |
| *Candidatus Desulforudis audaxviator* MP104C | Firmicutes |
| *Desulfitobacterium hafniense* DCP-2 | Firmicutes |
| *Desulfitobacterium hafniense* Y51 | Firmicutes |
| *Pelotomaculum thermopropionicum* SI | Firmicutes |
| *Syntrophobotulus glycolicus* FIGlyR, DSM 8271 | Firmicutes |
| *Thermincola potens* JR | Firmicutes |
| *Thermoanaerobacterium thermosaccharolyticum* DSM 571 | Firmicutes |
| *Thermoanaerobacter pseudethanolicus* 39E, DSM 33223 | Firmicutes |
| *Thermoanaerobacter* sp. X514 | Firmicutes |
| *Thermoanaerobacter italicus* Ab9, DSM 9252 | Firmicutes |
| *Thermoanaerobacter* *mathranii mathranii* A3, DSM 11426 | Firmicutes |
| *Thermoanaerobacter* sp. X513 | Firmicutes |
| *Thermoanaerobacter* *brockii* Ako-1, DSM 3389 | Firmicutes |
| *Thermoanaerobacter* *tengcongensis* MB4T | Firmicutes |
| *Caldicellulosiruptor lactoaceticus* 6A, DSM 9545 | Firmicutes |
| *Caldicellulosiruptor bescii* Z-1320, DSM 6725 | Firmicutes |
| *Caldicellulosiruptor saccharolyticus*, DSM 8903 | Firmicutes |
| *Caldicellulosiruptor obsidiansis* OB47 | Firmicutes |
| *Caldicellulosiruptor hydrothermalis* 108 | Firmicutes |
| *Caldicellulosiruptor owensensis* OL | Firmicutes |
| *Caldicellulosiruptor kristjanssonii* 177R1B, DSM 12137 | Firmicutes |
| *Caldicellulosiruptor kronotskyensis* 2002 | Firmicutes |
| *Moorella thermoacetica* ATCC 39073 | Firmicutes |
| *Carboxydothermus hydrogenoformans* Z-2901, DSM 6008 | Firmicutes |
| *Thermosediminibacter oceani* JW/IW-1228P, DSM 16646 | Firmicutes |
| *Mahella australiensis* 50-1 BON, DSM 15567 | Firmicutes |
| *Natranaerobius thermophilus* JW/NM-WN-LF | Firmicutes |
| *Halanaerobium hydrogeniformans* | Firmicutes |
| *Halanaerobium praevalens* GSL, DSM 2228 | Firmicutes |
| *Halothermothrix orenii* H 168 | Firmicutes |
| *Acetohalobium arabaticum* Z-7288, DSM 5501 | Firmicutes |
| *Acidaminococcus fermentans* VR4, DSM 20731 | Firmicutes |
| *Acidaminococcus intestini* RyC-MR95 | Firmicutes |
| *Veillonella parvula* Te3, DSM 2008 | Firmicutes |
| *Leptotrichia bruccalis* C-1013-b, DSM 1135 | Fusobacteria |
| *Sebaldella termitidis* ATCC33386 | Fusobacteria |
| *Ilyobacter polytropus* CuHBu1, DSM 2926 | Fusobacteria |
| *Fusobacterium nucleatum* ATCC 25586 | Fusobacteria |
| *Treponema pallidum* subsp. pallidum SS14 | Spirochaetes |
| *Treponema pallidum* subsp. pallidum Chicago | Spirochaetes |
| *Treponema succinifaciens* 6091, DSM 2489 | Spirochaetes |
| *Treponema azotonutricium* ZAS-9 | Spirochaetes |
| *Treponema primitia* ZAS-2 | Spirochaetes |
| *Treponema paraluiscuniculi* Cuniculi A | Spirochaetes |
| *Treponema pallidum* subsp. pallidum DAL-1 | Spirochaetes |
| *Treponema pallidum pertenue* CDC2 | Spirochaetes |
| *Treponema pallidum pertenue* Gauthier | Spirochaetes |
| *Treponema pallidum pertenue* SamoaD | Spirochaetes |
| *Treponema pallidum pallidum* Nichols | Spirochaetes |
| *Treponema denticola* ATCC 35405 | Spirochaetes |
| *Spirochaeta smaragdinae* SEBR 4228, DSM 11293 | Spirochaetes |
| *Spirochaeta thermophila* DSM 6192 | Spirochaetes |
| *Sphaerochaeta globosa* str. Buddy | Spirochaetes |
| *Sphaerochaeta pleomorpha* str. Grapes | Spirochaetes |
| *Sphaerochaeta coccoides* SPN1, DSM 17374 | Spirochaetes |
| *Brachyspira hyodysenteriae* WA1 | Spirochaetes |
| *Brachyspira pilosicoli* 95/1000 | Spirochaetes |
| *Brachyspira intermedia* PWS/A | Spirochaetes |
| *Brachyspira murdochii*  56-150, DSM 12563 | Spirochaetes |
| *Thermanaerovibrio acidaminovorans* Su883, DSM 6589 | Synergistetes |
| *Aminobacterium colombiense* ALA-1, DSM 12261 | Synergistetes |
| *Thermovirga lienii* Cas60314, DSM 17291 | Synergistetes |
| *Thermodesulfatator indicus* CIR 29812, DSM 15286 | Thermodesulfobacteria |
| *Thermotoga petrophila* RKU-1 | Thermotogae |
| *Thermotoga lettingae* TMO | Thermotogae |
| *Thermotoga* sp. RQ2 | Thermotogae |
| *Thermotoga neapolitana* DSM 4359 | Thermotogae |
| *Thermotoga naphthophila* RKU-10 | Thermotogae |
| *Thermotoga maritima* MSB8 | Thermotogae |
| *Thermosipho melanesiensis* BI429 | Thermotogae |
| *Thermosipho africanus* TCF52B | Thermotogae |
| *Fervidobacterium nodosum* Rt17-B1 | Thermotogae |
| *Opitutus terrae* PB90-1 | Verrucomicrobia |
| *Akkermansia muciniphila* ATCC BAA-835 | Verrucomicrobia |
| *Candidatus Cloacamonas acidaminovorans* | Not classified |
| *Candidatus Methylomirabilis oxyfera* | Not classified |
